# Methylation-based enrichment facilitates low-cost, noninvasive genomic scale sequencing of populations from feces

**DOI:** 10.1038/s41598-018-20427-9

**Published:** 2018-01-31

**Authors:** Kenneth L. Chiou, Christina M. Bergey

**Affiliations:** 1 Department of Anthropology, Washington University, St. Louis, MO 63130 USA; 2 Department of Psychology, University of Washington, Seattle, WA 98195 USA; 3 Department of Anthropology, New York University, New York, NY 10003 USA; 4 New York Consortium in Evolutionary Primatology, New York, NY USA; 5 Department of Anthropology, Pennsylvania State University, University Park, PA 16802 USA; 6 Department of Biology, Pennsylvania State University, University Park, PA 16802 USA

## Abstract

Obtaining high-quality samples from wild animals is a major obstacle for genomic studies of many taxa, particularly at the population level, as collection methods for such samples are typically invasive. DNA from feces is easy to obtain noninvasively, but is dominated by bacterial and other non-host DNA. The high proportion of non-host DNA drastically reduces the efficiency of high-throughput sequencing for host animal genomics. To address this issue, we developed an inexpensive capture method for enriching host DNA from noninvasive fecal samples. Our method exploits natural differences in CpG-methylation density between vertebrate and bacterial genomes to preferentially bind and isolate host DNA from majority-bacterial samples. We demonstrate that the enrichment is robust, efficient, and compatible with downstream library preparation methods useful for population studies (e.g., RADseq). Compared to other enrichment strategies, our method is quick and inexpensive, adding only a negligible cost to sample preparation. In combination with downstream methods such as RADseq, our approach allows for cost-effective and customizable genomic-scale genotyping that was previously feasible in practice only with invasive samples. Because feces are widely available and convenient to collect, our method empowers researchers to explore genomic-scale population-level questions in organisms for which invasive sampling is challenging or undesirable.

## Introduction

The past decade has witnessed a rapid transformation of biological studies with the continuing development and adoption of massively parallel sequencing technology. This sequencing revolution, however, has thus far had a relatively muted impact on studies of wild nonmodel organisms due largely to the difficulty of obtaining high-quality samples. This problem is particularly salient for endangered animals, cryptic animals, or animals for which it is otherwise difficult, undesirable, or unethical to obtain samples invasively.

Field researchers working with wild animals have explored several noninvasive sample types for DNA analysis including feces, hair, urine, saliva, feathers, skin, and nails^1^. Of these, feces may be the most readily available in many taxa^2^. Indeed, since PCR amplification of DNA from feces was first demonstrated in the 1990s^3^, noninvasive genetic studies from feces have revolutionized our understanding of the evolution, population structure, phylogeography, and behavior of nonmodel organisms. PCR amplification, however, is effective only for short sequences of DNA. The ability to generate cost-effective genomic-scale data of animals from feces using massively parallel sequencing would therefore constitute an important methodological advance towards bringing a greater number of wild organism studies into the genomic age.

Feces presents significant challenges for genetic analysis. DNA in feces is often fragmented and low in quantity. Fecal DNA extractions are further characterized by a frequent presence of co-extracted PCR inhibitors, sometimes complicating PCR detection of genotypes^1^, particularly with long amplicons. Finally, endogenous (host) DNA in feces constitutes a very low proportion, typically less than 5%^4–6^, of total fecal DNA. Instead, fecal DNA contains a preponderance of DNA from exogenous (non-host) sources such as gut microbes, digesta, intestinal parasites, and environmental organisms. Gut bacteria pose a particular challenge as they account for the highest proportion of DNA in feces^4,5^.

Because of the high representation of exogenous DNA in feces, shotgun sequencing of fecal DNA would yield only a small proportion of reads matching the host genome. For genomic studies of host organisms, particularly those targeting populations, this represents a crippling obstacle in the presence of typical financial constraints. Without an effective enrichment procedure, sequencing of fecal DNA would be less efficient than that of invasively obtained “high-quality” DNA by at least one order of magnitude regardless of improvements in sequencing throughput or cost.

Attempts to enrich host DNA from feces for genomic analysis^5,6^ have thus far employed targeted sequence capture methodologies. Sequence capture, like PCR, enriches DNA based on sequence specificity but unlike traditional PCR can work at any scale from a single locus^7^ to a whole genome^6,8,9^. This method involves hybridizing DNA or RNA “baits,” either affixed to an array^10,11^ or to magnetic beads in solution^12^, to a mixture of target and nontarget sequences, thereby capturing targeted DNA from the mixture. Sequence capture has been used for instance to enrich human exomes^13^, reduced-representation genomes^14–16^, host DNA from ancient or museum specimens^9,17–19^, and pathogen genomes from human clinical samples^8^. While the cost of custom oligonucleotide bait synthesis remains high, methods for transcribing custom baits from existing DNA templates^8,9^ have driven costs significantly down, increasing sequence capture’s appeal.

Perry *et al*.^5^ first successfully enriched host DNA from feces at the genomic scale. Using a modified sequence capture employing custom-synthesized baits, they were able to highly enrich 1.5 megabases of chromosome 21, the X chromosome, and the mitochondrial genome from fecal samples of 6 captive chimpanzees. Their protocol, however, remains prohibitively expensive for population-level analysis due to the high cost of bait synthesis. More recently, Snyder-Mackler *et al*.^6^ performed whole-genome capture on fecal DNA, using RNA baits transcribed *in vitro* from high quality baboon samples to enrich host genomes from 62 wild baboons. Resulting libraries were sequenced to low coverage (mean 0.49×), but nevertheless provided sufficient information for reconstructing pedigree relationships.

Despite these methodological advances, targeted sequence capture has distinct drawbacks. To avoid the high cost of bait synthesis, RNA baits must first be transcribed from high-quality genomic DNA that is consumed by the process, limiting its appeal when working with species for which high-quality DNA is difficult to obtain or in short supply. The processes of both bait generation and hybridization with fecal DNA are labor-intensive and time-consuming, with the hybridization including an incubation step that alone takes 1–3 days^6^. Because both RNA baits and the gDNA used to transcribe them are eventually depleted, the composition of RNA baits varies between bait sets, potentially impeding comparison of samples sequenced using different RNA baits and gDNA templates. *Trans* genomic captures (*i*.*e*. capturing DNA using baits from a different species) may complicate enrichment and introduce at least some capture biases^20^, which will be a particular impediment for genomic studies for which high-quality DNA from related taxa is not accessible. Sequence capture may also introduce biases toward the capture of low-complexity, highly repetitive genomic regions, as well as an excess of fragments from the mitochondrial genome^6,9,21^.

**Figure 1:**
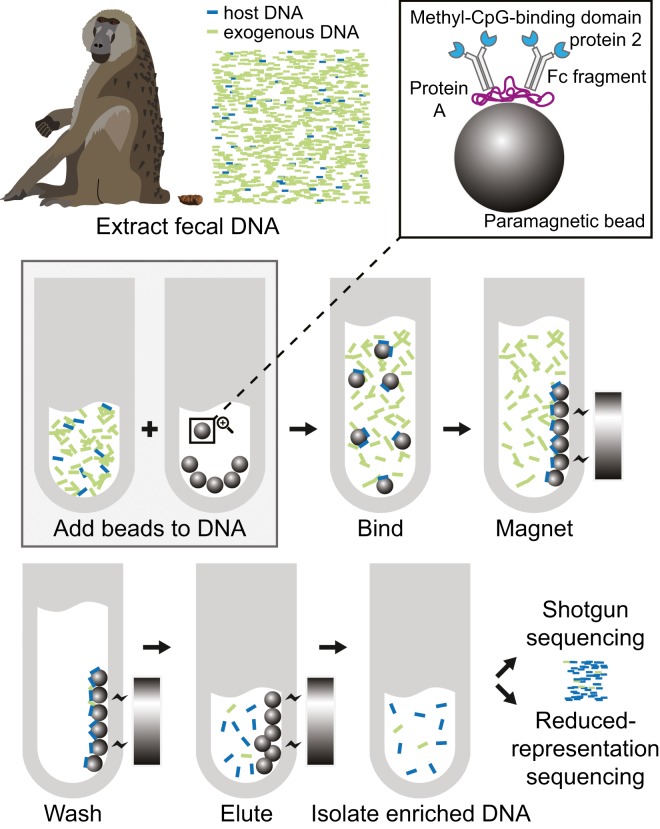
Overview of the FecalSeq method.

We have developed a method that makes noninvasive population genomics economically and practically feasible for the first time, by exploiting natural, evolutionarily ancient differences in CpG-methylation densities between vertebrate and bacterial genomes to enrich the host genome from feces. This method, which we call FecalSeq, uses methyl-CpG-binding domain (MBD) proteins to selectively bind and isolate DNA with high CpG-methylation density. Modified after techniques to enrich the microbiome from vertebrate samples^22^, our method employs a bait protein created by genetically fusing the human methyl-CpG binding domain protein 2 (MBD2) to the Fc tail of human IgG1. The resulting MBD2-Fc protein is then bound by a paramagnetic Protein A immunoprecipitation bead to create a complex that selectively binds double-stranded DNA with 5-methyl CpG dinucleotides. Because vertebrate DNA contains a high frequency of methylated CpGs^23,24^ while bacterial DNA does not^25,26^, this MBD bait complex selectively binds host DNA (Fig. 1). This enrichment method is inexpensive and, crucially, captures target DNA without modification, thereby enabling downstream library preparation techniques including complexity reduction-based sequencing methods such as RADseq, which we validate in this study by preparing and sequencing double-digest RADseq libraries^27^. Because of these properties, our method is well-suited for population genomic studies requiring high sequencing coverage, including those of nonmodel organisms for which few resources (e.g., high-quality samples or reference genomes) exist.

## Results

Our enrichment approach captures eukaryotic DNA using a methylated CpG binding domain protein fused to the Fc fragment of human IgG (MBD2-Fc) to selectively target sequences with high CpG methylation density^22^.

To evaluate our approach, we enriched DNA extractions from the feces of 6 captive and 46 wild baboons, which we then used to prepare and sequence ddRADseq libraries. We also prepared ddRADseq libraries from blood-derived genomic DNA of all six captive baboons to facilitate controlled (same-individual) comparisons of blood and fecal libraries. All libraries were sequenced using Illumina sequencing.

Quantitative PCR estimates of starting host DNA proportions in fecal DNA extracts ranged widely, but were substantially lower in samples obtained from the wild (captive samples: mean 5.3%, range <0.01–17.4%; wild samples: mean 0.6%, range <0.01–4.9%; Supplemental Tables S1–S2).

Based on two pilot libraries constructed from MBD-enriched fecal DNA, we found that there was large variation in the proportion of reads mapping to the baboon reference genome (mean 24.8%, range 0.7–81.2%; Supplemental Fig. S1; Supplemental Table S3), with the read mapping proportion correlating with starting host DNA proportions as estimated via qPCR (library A: *r*^2^ = 0.7338; *p* = 0.03; library B: *r*^2^ = 0.9127, *p* < 0.01). Endogenous DNA proportions on average increased 13-fold as estimated via comparison of pre-enrichment host proportion (from qPCR) and post-enrichment proportion of reads mapped (range 4.4–29.6; two samples removed due to starting proportions too low to quantify).

While some samples in our pilot libraries had high host DNA proportions following enrichment, these samples tended to already have high host DNA proportions prior to enrichment. Host DNA proportions following enrichment in the pilot libraries averaged only 4%, for instance, when samples with starting host DNA proportions greater than 1% were excluded. Because wild fecal DNA samples in our dataset on average started with less than 1% host DNA, we undertook a series of protocol optimization experiments to maximize the enrichment of these “low-quality” samples (Supplemental Tables S4–S7).

Using a revised protocol based on our optimization experiments (Supplemental Protocol), we created and sequenced a third library from MBD-enriched fecal DNA. After noting substantial improvements in enrichment, we finally sequenced a fourth library with MBD-enriched fecal DNA from 40 wild baboons.

Despite having similar or even lower starting host DNA proportions, read mapping proportions in the third library were substantially higher than the prior two (mean 49.1%, range 8.9–75.3%; Fig. S3; Supplemental Table S3). Endogenous DNA proportions on average increased 318-fold (range 4.3–2632.2; one sample removed due to starting proportion too low to quantify).

The fourth library consisting entirely of fecal DNA from wild animals had the lowest starting concentrations of host DNA (mean 0.3%, range < 0.01–3.1%). Following enrichment, however, host DNA proportions were nonetheless higher than our pilot libraries (mean 28.8%, range 1.5–73.6%; Supplemental Fig. S1; Supplemental Table S3). Endogenous DNA proportions on average increased 195-fold (range 23.7–486.9).

**Figure 2:**
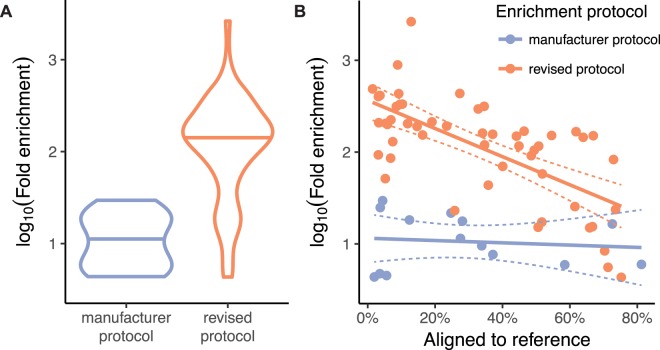
Comparison of the enrichment magnitude using the manufacturer protocol and the revised protocol. (**A**) Violin plots with the mean depicted show that the revised protocol results in substantially higher fold enrichment by approximately one order of magnitude. (**B**) A scatter plot shows that the revised protocol is particularly effective for samples with low starting quantities of host DNA. While some samples still had relatively small percentages of reads mapping to the baboon reference genome, these generally also exhibited the highest fold increases.

Overall, the revised protocol produced substantially higher enrichment, measured as fold increases in the proportion of host DNA, particularly for samples with very low starting proportions of host DNA (Fig. 2). While we sometimes were forced to use multiple rounds of extraction, thereby introducing variation in starting host proportions across same-individual trials, the revised protocol nonetheless exhibited robust improvement in read mapping proportions even when starting host proportions were substantially lower.

The distribution of blood- and fecal-derived reads did not differ significantly in the length of RADtags, the GC percentage, or the local CpG density, defined as the number of CpG sites in a region ± 5,000 bp from the boundaries of a RADtag (Wilcoxon rank sum tests, *p* > 0.99 for all three tests; Supplemental Fig. S2).

**Figure 3:**
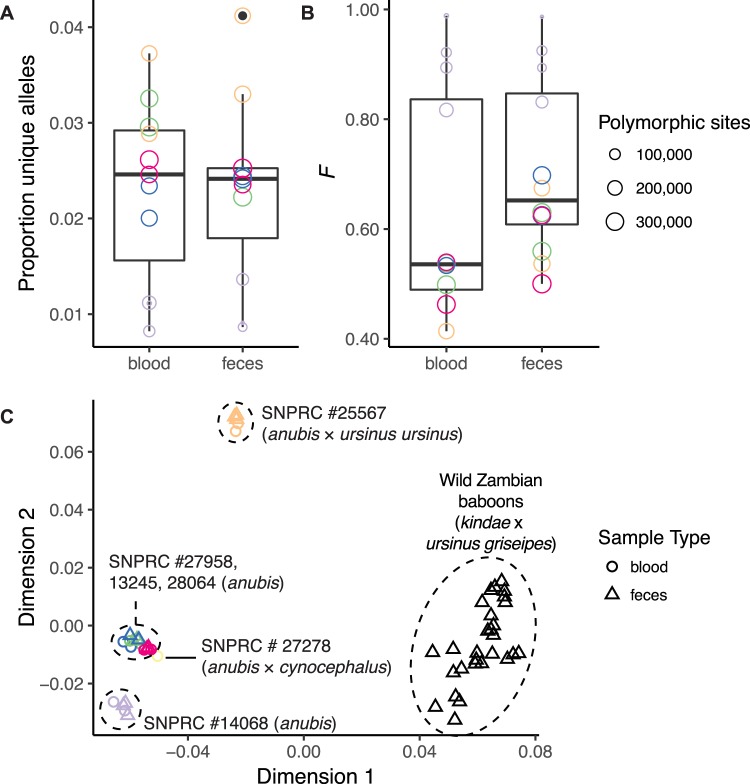
Concordance between blood- and feces-derived genotyping data from the same individuals. Colors symbolize the six captive individuals included in our study. Within these individuals, we did not find significant differences in (**A**) the proportion of unique alleles or (**B**) inbreeding coefficients from blood- and feces-derived libraries. The multidimensional scaling plot of identity-by-state shows (**C**) population structuring concordant with the known ancestry of animals (Supplemental Table S1). Distances between feces- and blood-derived sets of genotypes from the same individual are minimal, indicating that noise added by the enrichment method is dwarfed by the population structure signal in this baboon population dataset.

MBD binding may in principle select for genomic regions with relatively high CpG-methylation density, leading to dropout of other loci. Assessment of the concordance between blood- and feces-derived reads from the same individual was complicated by the correlation in ddRADseq between total reads and expected RADtags recovered and thereby SNPs discovered: a given RADtag is sequenced at a frequency inversely proportional to the deviation of its length from the mean of the size selection. Thus, we had to discern between dropout due to coverage-related stochasticity inherent in ddRADseq^27^ and that due to MBD enrichment. To perform this comparison, we computed the proportion of unique alleles between blood- and feces-derived RADtags from the same individual. For this test, we controlled for variation in sequencing coverage by randomly sampling reads as necessary in order to equalize total coverage among same-individual samples. Allelic dropout due to MBD enrichment would result in a higher proportion of alleles unique to blood-derived libraries relative to feces-derived libraries. We did not find a significant discrepancy (multi-sample-called SNPs: mean proportion unique alleles in blood = 2.3%, mean proportion unique alleles in feces = 2.3%; Wilcoxon signed rank test, *p* = 0.97; Fig. 3A).

Dropout of entire RADtags is easily detectable given a reference genome or sufficient samples for comparison; dropout of a single allele at heterozygous sites is a more insidious potential bias. Allelic dropout due to MBD enrichment would result in a decrease in heterozygosity in MBD-enriched fecal libraries. Inbreeding coefficients (*F*) computed from same-individual RADtags exhibited in some cases higher values for feces-derived samples (Fig. 3B). This difference, however, was not statistically significant (mean *F*_blood_ = 0.63; mean *F*_feces_ = 0.71; Wilcoxon signed rank test, *p* = 0.47), indicating low allelic dropout attributable to the MBD enrichment. For this test, we also controlled for variation in sequencing coverage as described above.

As investigations of population structure are one potential application of our method, we visualized the wild and captive baboons’ identity-by-state via multidimensional scaling (MDS) using PLINK^28,29^, and confirmed that individuals clustered by their known species or ancestry and that blood- and feces-derived reads from the same individual were close together in the MDS space (Fig. 3C). The results of this “sanity check” are unsurprising, as variance in samples encapsulated by the first MDS components is expected to reflect population and species membership.

Stringent filtration of SNP sets, as would be implemented in a standard population genetic study, reduced the apparent biases attributable to fecal enrichment, measured both as total SNPs with a significant association with sample type (unfiltered: 25,079 out of 591,726, or 4.2%; filtered: 13 out of 7,202, or 0.2%) as well as total SNPs with significant missingness assessed via a chi-square test (unfiltered: 69,753 out of 550,224, or 12.7%; filtered: 0 out of 5,602, or 0%). Though more work is needed to quantify more exactly the extent and causal factors that lead to missingness, many population genetic analyses are robust to the low level of dropout our analyses reveal in addition to that which is inherent in the RADseq family of techniques^30^.

## Discussion

Our methylation-based capture method achieves substantial enrichment of host DNA from fecal samples. Using our revised protocol developed through experimentation, we produced a mean 195-fold enrichment on our final library consisting entirely of fecal DNA obtained noninvasively under remote field conditions, with most samples nearly a decade old. A mean 28.8% of reads mapped to the baboon genome, despite starting with only a mean 0.34% of host DNA. Using fecal and blood DNA obtained from captive animals, we further demonstrate that feces-derived genotyping data following our method are concordant with corresponding data obtained from blood.

Feces are among the most readily accessible sources of information on wild animals^1^, and are particularly useful for population-level studies or studies of endangered or elusive species for which obtaining high-quality samples is difficult or undesirable. By exploiting methylation differences rather than sequence differences between host and bacterial DNA, FecalSeq is an enrichment strategy that requires neither prior genome sequence knowledge nor the use of high-quality DNA for preparation of capture baits. This results in enrichment which is both inexpensive and replicable. The enrichment procedure is also relatively rapid and uncomplicated. Using a 96-well plate, we performed two sequential rounds of enrichment on all forty samples in our final library within a day (see Supplemental Protocol).

Compared to comparable experiments using high-quality DNA samples such as blood, our enrichment method introduces extremely low added costs. After excluding shared costs such as DNA extraction, library preparation, and sequencing, major costs associated with our method are qPCR reagents for initial quality assessment of fecal DNA samples and enrichment reagents for capturing the host genome. qPCR reagents cost about $0.60 USD per reaction (or $1.20 per sample assuming samples are run in duplicate). For our enrichment protocol, the amount of reagents used will vary based on the starting proportion of host DNA in the sample (see Supplemental Protocol). Assuming fecal DNA samples on average contain 2.5% host DNA, a single enrichment kit will support a total of 240 enrichment reactions at $0.70 per sample. Based on our experience, most fecal DNA samples contain less than 2.5% host DNA and will therefore require less reagents, further lowering the cost per sample. Following enrichment, we purified DNA using homemade SPRI beads^31^ which add a very low cost per sample (about $0.10 per sample).

Altogether, compared to high-quality DNA experiments, the marginal cost of our method is approximately an additional $2 USD per sample factoring in duplicate qPCR reactions, enrichment, and SPRI bead cleanup. These costs represent a substantial decrease relative to enrichment methods based on oligonucleotide-based sequence capture^5,6^, which at present represents the only other published strategy for enriching fecal DNA. Snyder-Mackler *et al*.^6^ present the most cost-effective sequence-capture-based strategy to date at a cost of $60 per sample, or $29 per sample when using a multiplexed capture strategy on 10 samples at a time. Overall, our strategy therefore decreases the cost of enrichment by about one order of magnitude.

Importantly, FecalSeq is to our knowledge the first genomic-scale fecal DNA enrichment method that is compatible with most downstream library preparation methods for massively parallel sequencing. Through our use of ddRADseq, we demonstrate that our method facilitates low-cost high-capacity genotyping of wild populations without introducing significant bias. Further, because ddRADseq is customizable^27^, there is substantial flexibility for researchers to optimize the number of samples and the fraction of the genome sequenced for particular research questions. This is not possible for libraries prepared using targeted sequence capture, which are therefore currently limited mainly to low-coverage analyses at the population level^6^. Transcription of sequence capture baits from reduced-representation libraries may potentially help address this problem^14–16^, but its efficacy for fecal DNA has yet to be demonstrated.

Although ideally suited to taxa with a reference genome, genotyping via double-digest RADseq is possible for species that lack a closely related reference genome. We aligned our sequencing reads to the baboon reference genome for this study, but our approach is likely also applicable to species without a reference genome. A reference genome from a more phylogenetically distant species can be used, although reads from divergent regions will fail to map. If a nearby genome is not available, an additional pre-screening step would be necessary, in which exogenous reads are filtered out through comparison to the nearest available genome, before proceeding to clustering and variant identification as per normal reference-free ddRADseq methods. Although these caveats will be mitigated as more genomes become available, the proximity of study individuals to an available reference genome is an important consideration when deciding if ddRADseq methods, including ddRADseq following our enrichment method, are suitable to address the research goals.

We robustly found that sequencing efficiency (percentage of reads assigned to target genome) of MBD-enriched fecal DNA libraries correlates strongly with starting proportions of host DNA, echoing findings using other capture methods^6^. Future attention should therefore be directed towards fecal sample collection, storage, and extraction methods that maximize the selective recovery of host nuclear DNA^32^. While we demonstrated effective genotyping of samples with often very low starting proportions of host DNA (the vast majority < 0.5%), future studies may consider pre-screening extracted DNA samples using qPCR to select for samples with high starting proportions of host DNA.

We found that, for unknown reasons, starting host DNA concentration for fecal samples collected in captive conditions was higher than that of fecal samples collected from the wild, despite consistent sample collection and processing protocols. We speculate that these differences may be due to either the freshness of samples collected in captivity or to dietary differences between captivity and the wild. Because our wild fecal samples were collected under common biological field conditions (e.g., tropical heat and humidity, lack of refrigeration), our study underscores the need to validate enrichment methods for wild animals using samples from the wild.

Low starting proportions of host DNA present a challenge not only because they result in lower sequencing efficiency, but also because they correlate with low absolute quantities of DNA belonging to the host organism. In some cases, particularly in samples collected from wild animals under field conditions, starting proportions of host DNA were so low that only approximately 0.1 ng of target DNA was available in a 1 μg fecal DNA extract. Given the large genome sizes of baboons (approximately 3 Gb) and many other vertebrates, substantial allelic dropout is expected in these cases. Significantly, this challenge cannot be fully addressed by this or any other enrichment method and remains an important consideration for researchers working with feces. It can be minimized, however, by optimizing the enrichment procedures to maximize the recovery of target DNA present in a fecal DNA sample, as well as by increasing the total amount of starting fecal DNA.

Because MBD enrichment partitions DNA based on CpG-methylation density, FecalSeq does not enrich hypomethylated host mitochondrial DNA^33^. While this may be undesirable for studies requiring the matrilineally inherited marker, it also precludes the disproportionately high representation of mitochondrial DNA that is typical in libraries prepared using the targeted sequence capture approach^5,6,9,21^. FecalSeq may, however, co-enrich nuclear DNA from exogenous eukaryotes such as from plant or animal digesta. Care should therefore be taken to minimize the presence of exogenous eukaryotic tissues or cells, although the degree to which this is a problem in practice is currently unknown. As cell-wall-bound plant cells may be more likely to pass through the digestive tract intact, extraction methods that minimize lysis of cell walls should be preferred. We speculate that prey DNA from carnivorous animals may be more difficult to partition from host DNA. While our comparisons of inbreeding coefficients, unique alleles, and locus-by-locus statistical tests from matched blood and fecal samples revealed no significant effect of sample type and therefore enrichment, our small sample size limits our power to detect differences with small effect size. Subsequent studies with larger sample sizes may detect a significant effect of enrichment, but one which may be negligible or correctable for some applications.

Since PCR amplification of DNA from feces was first achieved in the 1990s^3,34,35^, noninvasive genetic studies have revolutionized our understanding of the evolution, ecology, and behavior of nonmodel organisms. By facilitating low-cost genomic-scale sequencing from feces, our method connects a community of field researchers with the benefits of massively parallel sequencing, ushering noninvasive organism studies into the genomic age.

## Methods

### Samples

Blood and fecal samples were collected from six captive baboons (genus *Papio*) housed at the Southwest National Primate Research Center (SNPRC) at the Texas Biomedical Research Institute. The individuals were of either *P*. *anubis* or hybrid ancestry (Supplemental Table S1). All six baboons were fed a diet manufactured by Purina LabDiet (“Monkey Diet 15%”) containing 15% minimum crude protein, 4% minimum crude fat, and 10% maximum crude fiber. In separate sedation events, blood and feces were collected from the same individual who was isolated for the duration of the sedation. Following centrifugation, the buffy coat was isolated from blood samples and stored at −80 °C. 2 ml of feces were also collected into 8 ml tubes containing 4 ml of RNALater (Ambion).

In addition, we collected or obtained fecal samples from 46 wild baboons in Zambia. Samples were collected between 2006 and 2015 from the Luangwa Valley, the Lower Zambezi National Park, Choma, or Kafue National Park and are of *P*. *kindae* × *P*. *cynocephalus*, *P*. *ursinus griseipes*, or *P*. *kindae* × *P*. *ursinus griseipes* ancestry (Supplemental Table S1). As with the SNPRC samples, 2 ml of feces were collected into 8 ml tubes containing 4 ml of RNALater. In contrast to the SNPRC samples, however, these samples were collected noninvasively from unhabituated animals in remote field conditions. Samples therefore could not be attributed to particular animals, although samples were selected to avoid duplication using either field observations or geographic distance. Following collection, samples were stored without refrigeration for 1–6 months before being frozen at −20 °C for long-term storage.

All procedures involving live animals were carried out in accordance with relevant guidelines and regulations. Experimental procedures at SNPRC were conducted with approval by the Institutional Animal Care and Use Committee of the Texas Biomedical Research Institute (protocol #1403 PC 0). Sedation and blood draws were performed under the supervision of a veterinarian and animals were returned immediately to their enclosures following recovery. Sample collection in Zambia was conducted with approval by the Animal Studies Committee of Washington University (assurance #A-3381–01) and following local laws and regulations in Zambia.

Buffy coat extractions were performed using the QIAamp DNA Blood Mini Kit (Qiagen), following manufacturer’s instructions. Fecal extractions were performed using the QIAamp DNA Stool Mini Kit (Qiagen) following manufacturer’s instructions for optimizing host DNA yields. DNA concentration and yield were measured using a Qubit dsDNA BR Assay (Life Technologies). In some cases, multiple DNA extractions from the same individuals were necessary when DNA was depleted over the course of this study.

We estimated the proportion of host DNA for each fecal DNA extraction using quantitative PCR (qPCR) by comparing estimates of host DNA concentration obtained by qPCR to estimates of total fecal DNA concentration obtained by Qubit. Amplification was conducted using universal mammalian *MYCBP* primers^36^ and evaluated against a standard curve constructed from the liver DNA of an individual baboon. Samples and standards were run in duplicate alongside positive and negative controls (see Supplemental Protocol for full details).

### DNA enrichment

DNA was enriched using the NEBNext Microbiome DNA Enrichment Kit (New England Biolabs)^22^.

MBD2-Fc-bound magnetic beads were prepared according to manufacturer instructions in batches ranging from 40 to 160 μl. For each *n* μl batch, we prebound 0.1 × *n* μl MBD2-Fc protein to *n* μl protein A magnetic beads by incubating the mixture with rotation for 10 min at room temperature. The bound MBD2-Fc magnetic beads were then collected by magnet and washed twice with 1 ml ice-cold 1x bind/wash buffer before being resuspended in *n* μl ice-cold 1x bind/wash buffer.

As a pilot experiment, we prepared two successive libraries, library A and library B, following manufacturer’s instructions for capturing methylated host DNA, with minor protocol modifications incorporated for the second pilot library (library B). Library A included MBD-enriched fecal DNA from 4 SNPRC baboons and 2 Luangwa Valley baboons, as well as blood DNA from the same SNPRC baboons. Library B included MBD-enriched fecal DNA from 4 SNPRC baboons (with two repeats from library A), 4 Kafue National Park baboons, and 2 Luangwa Valley baboons, as well as blood DNA from 2 SNPRC baboons. For each fecal DNA sample, we combined 1–2 μg of extracted fecal DNA with 160 μl of prepared protein-bound beads and a variable volume of ice-cold 5x bind/wash buffer for maintaining 1x concentration of bind/wash buffer. After combining beads and DNA, we incubated the mixture at room temperature with rotation for 15 min. DNA and MBD2-Fc-bound magnetic beads were then collected by magnet and the supernatant removed. For samples in library A, we washed the collected beads with 1 ml of ice-cold 1x bind/wash buffer. For samples in library B, we conducted three expanded wash steps to maximize the removal of unbound DNA. For each wash in library B, we added 1 ml of ice-cold 1x bind/wash buffer and mixed the beads on a rotating mixer for three minutes at room temperature before collecting the beads by magnet and removing the supernatant. Following the final wash, we resuspended and incubated the beads at 65 °C with 150 μL of 1x TE buffer and 15 μL of Proteinase K for 20 min with occasional mixing. The eluted DNA was then separated by magnet, purified with 1.5x homemade SPRI bead mixture^31^, and quantified using a Qubit dsDNA HS Assay (Life Technologies).

Our pilot sequencing results from libraries A and B revealed large variation in the percentage of reads mapping to the baboon genome, with mapping percentages ranging from 1.1% to 79.3%, with much of the variation correlating with the proportion of host DNA in the unenriched fecal DNA sample (Supplemental Fig. S3). To expand the utility of the enrichment protocol to all fecal DNA samples, we conducted a series of capture experiments designed to optimize the enrichment of host DNA from “low-quality” samples (i.e., samples with low proportions of host DNA). For these experiments, we artificially simulated fecal DNA by combining high-quality baboon liver or blood genomic DNA (liver: SNPRC ID #19334; blood: SNPRC ID #14068 or #25567) with *E*. *coli* DNA (K12 or ATCC 11303 strains) at controlled proportions. The resulting post-enrichment proportion of baboon and *E*. *coli* DNA was evaluated by qPCR in two analyses using (1) universal mammalian *MYCBP*^36^ and (2) universal bacterial 16 S rRNA (16 S)^37^ primers along with standards created from the same respective organisms (experiments and results are described in detail in Supplemental Table S2).

Based on these capture optimization experiments, we prepared subsequent libraries using a version of the protocol incorporating modifications demonstrated to improve enrichment. Despite preferentially binding CpG-methylated DNA, the MBD2-Fc bait complex nevertheless bound a fraction of nonmethylated DNA. We therefore aimed to minimize both the absolute and relative amount of nonmethylated DNA binding to the bead complex. In our tests, the amount and fraction of nonmethylated bound DNA was highest when the ratio of the MBD2-Fc magnetic bead complex to total DNA was high, suggesting a surplus of MBD2 binding sites given the relatively small fraction of CpG-methylated DNA. We therefore reduced the ratio of prepared MBD2-Fc-bound magnetic beads to total DNA by tuning the amount of beads to the estimated amount of CpG-methylated host DNA. Because the amount of CpG-methylated host DNA in feces is extremely low, this modification also greatly decreased the cost of the reagents. Through our optimization experiments, we found that incorporating an additional wash step reduced the amount of contaminating nonmethylated DNA captured. Finally, we developed a method for serial enrichment of the samples (repeating the enrichment protocol), which substantially improved results. Our initial serial enrichment experiments failed to recover DNA, likely due to the use of proteinase K (combined with TE buffer) in the manufacturer’s elution protocol. Hypothesizing that incomplete removal of proteinase K, even following bead cleanup, interfered with the enrichment protocol, we instead eluted DNA using a high (2 M) NaCl concentration, resulting in successful serial enrichment. These changes, along with a full modified protocol, are detailed in the Supplemental Protocol.

For our next two libraries, libraries C and D, we added a much smaller volume of prepared MBD2-Fc-bound magnetic beads (1–22 μl) based on the estimated proportion of starting host DNA, kept the capture reaction volume consistent at a relatively low 40 μl (concentrating samples as needed using a SPRI bead cleanup), added an extra wash step in which samples were resuspended in 100 μl of 1x bind/wash buffer then incubated at room temperature for 3 minutes with rotation, and eluted samples in 100 μl 2 M NaCl. For four fecal DNA samples in library C and all of library D, we serially enriched the samples by repeating the capture reaction with 30 μl of MBD-enriched DNA (post SPRI-bead cleanup). Library C included fecal DNA from 5 SNPRC baboons, 2 Kafue National Park baboons, and 1 Luangwa Valley baboon. Library D contained fecal DNA from 6 Lower Zambezi National Park baboons, 4 Choma baboons, and 30 Kafue National Park baboons. We prepared a final library, library E, from independently extracted blood DNA from five SNPRC baboons in order to quantify the stochasticity associated with independent library preparation from independent extracts. The composition of libraries A-E are described in detail in Supplemental Tables S2-S3.

### Library preparation and sequencing

Library preparation followed standard double-digest restriction site-associated DNA sequencing (ddRADseq) procedures^27^ with modifications to accommodate low input as described below.

For all samples, including blood DNA and MBD-enriched fecal DNA, we digested DNA with *Sph*I and *Mlu*CI (New England Biolabs), following a ratio of 1 unit of each enzyme per 20 ng of DNA. Enzymes were diluted up to 10× using compatible diluents (New England Biolabs) to facilitate pipetting of small quantities, using an excess of enzyme if necessary to avoid pipetting less than 1 μl of the diluted enzyme mix. As the total amount of post-enrichment fecal DNA is by nature low, we adjusted adapter concentrations in the ligation reaction to ~0.1 μM for barcoded P1 and ~3 μM for P2, which correspond to excesses of adapters between 1–2 orders of magnitude. Since adapter-ligated samples are multiplexed into pools in equimolar amounts, we made efforts to combine samples with similar concentrations and enrichment when known. We used the BluePippin (Sage Science) with a 1.5% agarose gel cassette for automated size selection of pooled individuals, with a target of 300 bp (including adapters) and extraction of a “tight” collection range (±39 bp). For PCR amplification, we ran all reactions in quadruplicate to minimize PCR biases and attempted to limit the number of PCR cycles. As the concentration of post-size-selection pools was below the limits of detection without loss of a considerable fraction of the sample, estimation of the required number of PCR cycles was difficult. We therefore iteratively quantified products post-PCR and added cycles as necessary. The total number of PCR cycles per pool is reported in Supplemental Table S3, but was usually 24. Finally, libraries were sequenced using either Illumina MiSeq (libraries A-C; 2 × 150 paired-end) or Illumina HiSeq. 2500 (library D; 2 × 100 paired-end) sequencing with 30% spike in of PhiX control DNA.

### Analysis

We demultiplexed reads by sample and mapped them to the baboon reference genome (Panu 2.0; Baylor College of Medicine Human Genome Sequencing Center) using BWA with default parameters and the BWA-ALN algorithm^38^. For every pair of blood and fecal samples from the same individual, we downsampled mapped reads to create new pairs with equal coverage in order to control for biases due to differences in sequencing depth. After realignment around indels, we identified variants using GATK UnifiedGenotyper^39^, in parallel analyses (1) calling variants in all samples at once and (2) processing each sample in isolation to avoid biasing variant calls from other samples at the expense of accuracy. Homozygous sites matching the reference genome were listed as missing when variants were inferred in single individuals. Variants were filtered with GATK VariantFiltration (filters: QD < 2.0, MQ < 40.0, FS > 60.0, HaplotypeScore > 13.0, MQRankSum < −12.5, ReadPosRankSum < −8.0) and indels were excluded.

We digested the baboon reference genome *in silico*, tallied reads within each predicted RADtag, and gathered the following information about each region: length, GC percentage, and CpG count in region ± 5 kb. We also calculated read depth in these simulated RADtags. Distributions of blood and fecal RADtags’ length, GC percentage, and local CpG density (Supplemental Fig. S2) were compared using Wilcoxon rank sum tests and visually inspected for possible gross distortion due to widespread dropout.

If the fecal enrichment procedure caused widespread allelic dropout, the proportion of alleles unique to the blood samples would be higher than that to the fecal sample. We tallied these unique alleles by using VCFtools^40^ to compute discordance on a per-site basis “--diff-site-discordance” as well as a discordance matrix “--diff-discordance-matrix”, and parsed the results to compute unique SNP percentage in paired blood and fecal samples. We tested for an excess of unique SNPs in blood with a Wilcoxon signed rank test.

To quantify loss of heterozygosity due to allelic dropout, we computed the method-of-moments inbreeding coefficient, *F* for all blood-feces pairs with equalized coverage, using both the individually called and multi-sample called SNP sets. *F* was calculated using the “--het” argument in PLINK^28,29^. The presence of dropout is expected to inflate *F*. We tested for differences in paired samples’ estimates of *F* via a Wilcoxon signed rank test. The dataset is not filtered for missingness, so sequencing errors inferred to be true variants may inflate heterozygosity estimates, thus deflating *F*.

To create a stringently filtered dataset with high genotyping rate, we filtered the multi-sample called SNPs in PLINK^28,29^, retaining only those genotyped in at least 90% of samples and removing samples with genotypes at fewer than 10% of sites. This filtered set was further pruned for linkage disequilibrium by sliding a window of 50 SNPs across the chromosome and removing one random SNP in each pair with *r*^2^ > 0.5. Using all samples, we performed multidimensional scaling to visualize identity by state (IBS). Using just the samples that were part of the same-individual blood-feces pairs, we then performed an association test and missingness chi-square test to detect allele frequencies or missingness that correlated with sample type. We did the same with the unfiltered dataset as well. Though we had few pairs of fecal samples from the same individual, we computed distance between pairs of samples from the same individual using the stringently filtered dataset to compare distance between and within sample types via a Wilcoxon signed rank test.

## Electronic supplementary material

Supplemental Information

## Data Availability

All raw sequencing reads are publicly accessible through the NCBI Sequence Read Archive (BioProject #369456, BioSample accessions #SAMN06286370 - SAMN06286448). All code generated for this project is released under a GNU v3.0 license, archived on Zenodo (doi:10.5281/zenodo.848299), and can be accessed at https://github.com/bergeycm/RAD-faex.
