# Supplementary material for: Methylation-based enrichment facilitates low-cost, noninvasive genomic scale sequencing of populations from feces
Source: Sci Rep. 2018 Jan 31;8:1975. doi: 10.1038/s41598-018-20427-9 (PMC5792461; doi:10.1038/s41598-018-20427-9)
Supplement: Supplementary file 1 — Supplemental Information [file 41598_2018_20427_MOESM1_ESM.pdf]

Title

Methylation-based enrichment facilitates low-cost, noninvasive genomic scale sequencing of populations from feces

Author list

Kenneth L. Chiou, Christina M. Bergey

Supplementary Information

Fig. S1. Percentage of reads mapping to the baboon reference genome (papAnu2) for all samples included in this study. Sixteen samples were enriched using the manufacturer protocol and 52 using the revised protocol.

Fig. S2. Combined distributions of (a) RADtag lengths, (b) CpG counts (within the boundaries of the sequenced RADtag ±5,000), and (c) GC percentages in sequenced libraries.

Fig. S3. Relationship between pre-enrichment host percentage, as estimated by quantitative PCR, and post-enrichment host percentage, as estimated by alignment of sequencing reads to the baboon reference genome.

Table S1. Animals sequenced for this study.

Table S2. Fecal DNA enrichment results.

Table S3. Library preparation and sequence mapping results.

Table S4. DNA samples used for controlled experiments.

Table S5. Controlled DNA enrichment experiments.

Table S6. Controlled DNA enrichment experiment results.

Table S7. Controlled DNA enrichment elution series.

Supplemental Protocol

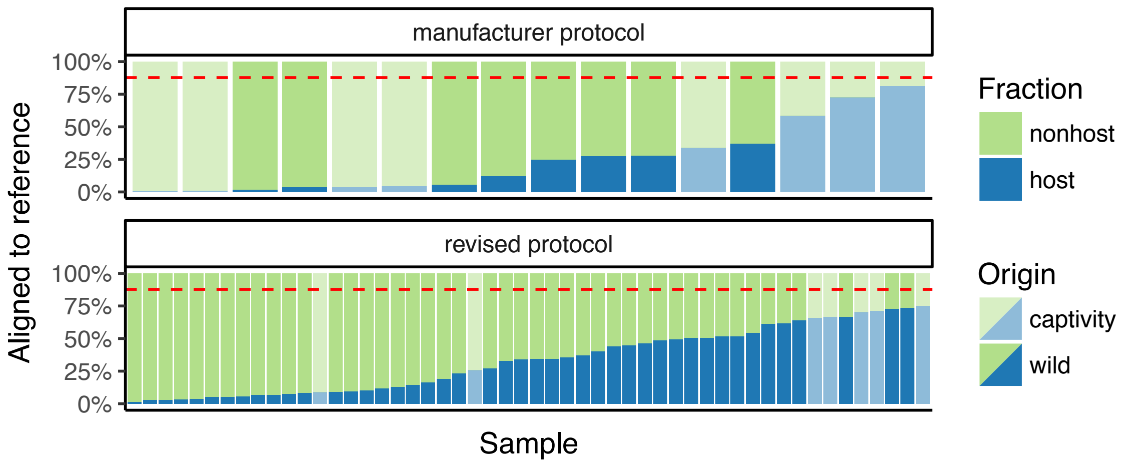

Supplemental Fig. S1: Percentage of reads mapping to the baboon reference genome (papAnu2) for all samples included in this study. Sixteen samples were enriched using the manufacturer protocol and 52 using the revised protocol.

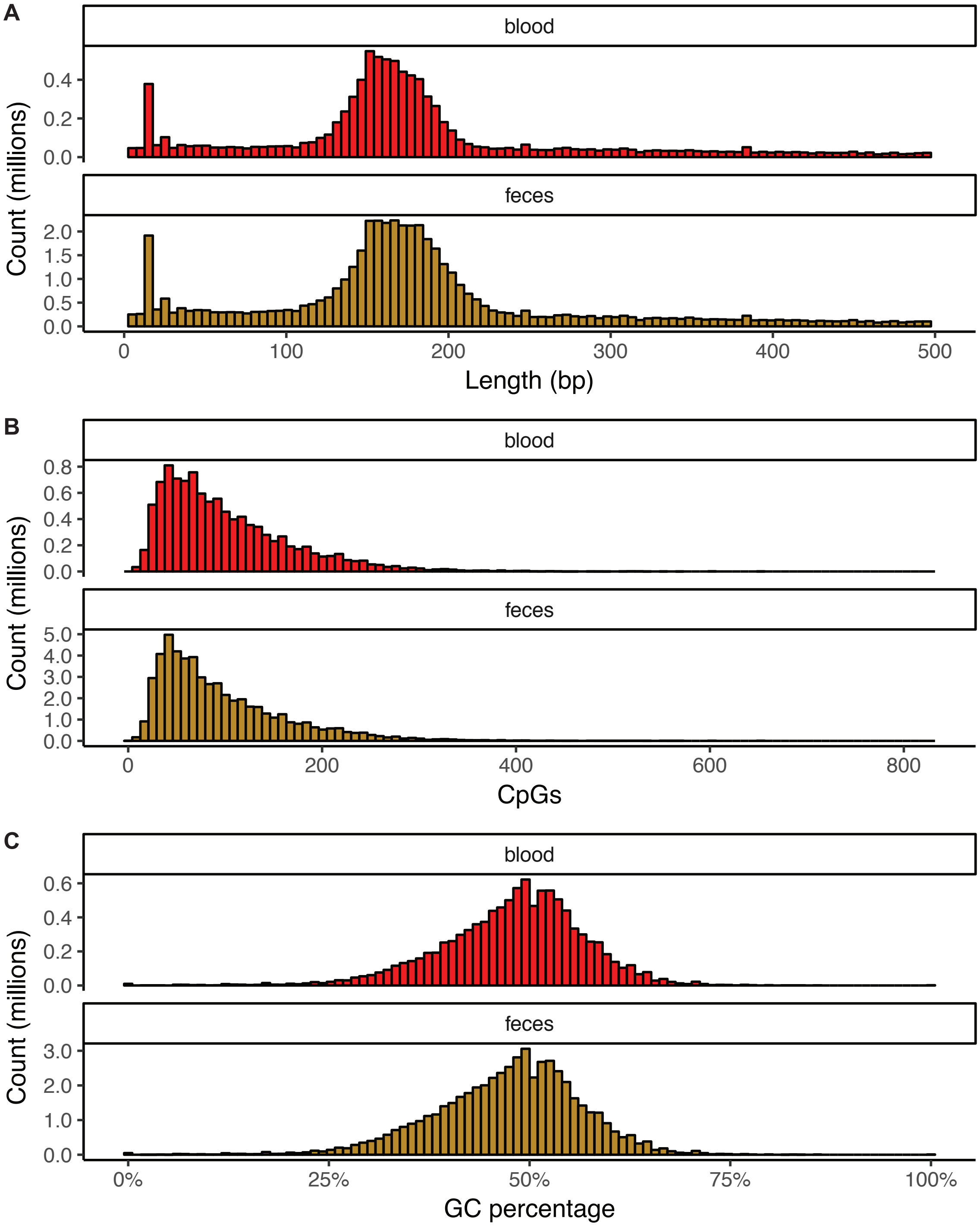

Supplemental Fig. S2: Combined distributions of (a) RADtag lengths, (b) CpG counts (within the boundaries of the sequenced RADtag $\pm 5,000$), and (c) GC percentages in sequenced libraries.

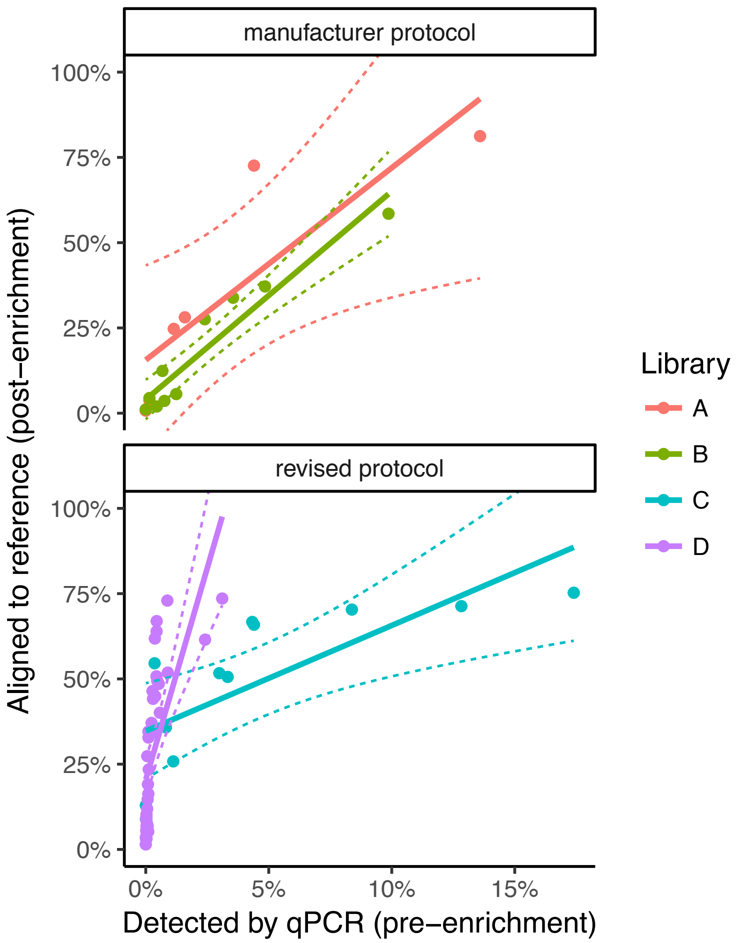

Supplemental Fig. S3: Relationship between pre-enrichment host percentage, as estimated by quantitative PCR, and post-enrichment host percentage, as estimated by alignment of sequencing reads to the baboon reference genome.

Supplemental Table S1: Animals sequenced for this study.

Individual	Locale	Taxon	Sex	Origin	Year
SNPRC #13245	SNPRC	*Papio anubis*	male	captivity	2014
SNPRC #14068	SNPRC	*Papio anubis*	male	captivity	2014
SNPRC #25567	SNPRC	*Papio anubis* × *Papio ursinus*	female	captivity	2014
SNPRC #27278	SNPRC	*Papio anubis* × *Papio cynocephalus*	male	captivity	2014
SNPRC #27958	SNPRC	*Papio anubis*	female	captivity	2014
SNPRC #28064	SNPRC	*Papio anubis*	female	captivity	2014
BZ06-051	South Luangwa NP	*Papio kindae* × *Papio cynocephalus*	unknown	wild	2006
BZ06-053	South Luangwa NP	*Papio kindae* × *Papio cynocephalus*	unknown	wild	2006
BZ06-066	South Luangwa NP	*Papio kindae* × *Papio cynocephalus*	unknown	wild	2006
BZ06-148	North Luangwa NP	*Papio kindae* × *Papio cynocephalus*	unknown	wild	2006
BZ06-218	Lower Zambezi NP	*Papio ursinus*	unknown	wild	2006
BZ06-220	Lower Zambezi NP	*Papio ursinus*	unknown	wild	2006
BZ06-221	Lower Zambezi NP	*Papio ursinus*	unknown	wild	2006
BZ06-224	Lower Zambezi NP	*Papio ursinus*	unknown	wild	2006
BZ06-225	Lower Zambezi NP	*Papio ursinus*	unknown	wild	2006
BZ06-227	Lower Zambezi NP	*Papio ursinus*	unknown	wild	2006
BZ07-001	Choma	*Papio ursinus*	unknown	wild	2007
BZ07-004	Choma	*Papio ursinus*	unknown	wild	2007
BZ07-005	Choma	*Papio ursinus*	unknown	wild	2007
BZ07-007	Choma	*Papio ursinus*	unknown	wild	2007
BZ07-029	Kafue NP	*Papio kindae* × *Papio ursinus*	unknown	wild	2007
BZ07-030	Kafue NP	*Papio kindae* × *Papio ursinus*	unknown	wild	2007
BZ07-032	Kafue NP	*Papio kindae* × *Papio ursinus*	unknown	wild	2007
BZ07-034	Kafue NP	*Papio kindae* × *Papio ursinus*	unknown	wild	2007
BZ07-035	Kafue NP	*Papio kindae* × *Papio ursinus*	unknown	wild	2007
BZ07-039	Kafue NP	*Papio kindae* × *Papio ursinus*	unknown	wild	2007
BZ07-041	Kafue NP	*Papio kindae* × *Papio ursinus*	unknown	wild	2007
BZ07-042	Kafue NP	*Papio kindae* × *Papio ursinus*	unknown	wild	2007
BZ07-045	Kafue NP	*Papio kindae* × *Papio ursinus*	unknown	wild	2007
BZ07-047	Kafue NP	*Papio kindae* × *Papio ursinus*	unknown	wild	2007
BZ07-100	Kafue NP	*Papio kindae* × *Papio ursinus*	unknown	wild	2007
Chiou-14-001	Kafue NP	*Papio kindae* × *Papio ursinus*	unknown	wild	2014
Chiou-14-003	Kafue NP	*Papio kindae* × *Papio ursinus*	unknown	wild	2014
Chiou-14-004	Kafue NP	*Papio kindae* × *Papio ursinus*	unknown	wild	2014
Chiou-14-005	Kafue NP	*Papio kindae* × *Papio ursinus*	unknown	wild	2014
Chiou-14-030	Kafue NP	*Papio kindae* × *Papio ursinus*	unknown	wild	2014
Chiou-14-036	Kafue NP	*Papio kindae* × *Papio ursinus*	unknown	wild	2014
Chiou-14-039	Kafue NP	*Papio kindae* × *Papio ursinus*	unknown	wild	2014
Chiou-14-041	Kafue NP	*Papio kindae* × *Papio ursinus*	unknown	wild	2014
Chiou-14-042	Kafue NP	*Papio kindae* × *Papio ursinus*	unknown	wild	2014
Chiou-14-044	Kafue NP	*Papio kindae* × *Papio ursinus*	unknown	wild	2014
Chiou-14-050	Kafue NP	*Papio kindae* × *Papio ursinus*	unknown	wild	2014
Chiou-14-054	Kafue NP	*Papio kindae* × *Papio ursinus*	unknown	wild	2014
Chiou-14-056	Kafue NP	*Papio kindae* × *Papio ursinus*	unknown	wild	2014
Chiou-14-057	Kafue NP	*Papio kindae* × *Papio ursinus*	unknown	wild	2014
Chiou-14-058	Kafue NP	*Papio kindae* × *Papio ursinus*	unknown	wild	2014
Chiou-14-059	Kafue NP	*Papio kindae* × *Papio ursinus*	unknown	wild	2014
Chiou-14-065	Kafue NP	*Papio kindae* × *Papio ursinus*	unknown	wild	2014
Chiou-14-069	Kafue NP	*Papio kindae* × *Papio ursinus*	unknown	wild	2014
Chiou-15-003	Kafue NP	*Papio kindae* × *Papio ursinus*	unknown	wild	2015
Chiou-15-004	Kafue NP	*Papio kindae* × *Papio ursinus*	unknown	wild	2015
Chiou-15-005	Kafue NP	*Papio kindae* × *Papio ursinus*	unknown	wild	2015

Supplemental Table S2: Fecal DNA enrichment results. Key: ID, capture experiment ID; Lib, library ID; Ind, individual (see Supplemental Table S1); PHB, percent host DNA before; TD, total fecal DNA used (ng); BV, bead volume used (μl); TV, total reaction volume (μl); TY, total DNA yield (ng); NE, number of enrichment steps; PHA, percent host DNA after; NDB, *n*-fold decrease in bacterial DNA.

ID	Lib	Ind	PHB	TD	BV	TV	TY	NE	PHA	NDB
A01F.T002	A	SNPRC #14068	0.15%	1,000.00	160.00	163.00	82.83	single	6.79%	-
A02F.T005	A	SNPRC #25567	4.40%	2,000.00	160.00	174.80	455.40	single	50.90%	-
A03F.T007	A	SNPRC #27278	0.00%	1,000.00	160.00	165.10	191.40	single	6.38%	-
A04F.T009	A	SNPRC #27958	13.59%	2,000.00	160.00	172.40	584.10	single	47.50%	-
A05F.B051	A	BZ06-051	1.59%	1,800.00	160.00	285.00	122.43	single	-	-
A06F.B053	A	BZ06-053	1.14%	1,365.00	160.00	535.00	119.79	single	-	-
B01F.T001	B	SNPRC #13245	3.55%	1,000.00	160.00	170.90	72.80	single	19.45%	-
B02F.T002	B	SNPRC #14068	0.15%	1,000.00	160.00	164.40	16.08	single	8.34%	-
B03F.T007	B	SNPRC #27278	0.00%	1,000.00	160.00	165.40	26.80	single	12.68%	-
B04F.T010	B	SNPRC #28064	9.87%	1,000.00	160.00	177.50	78.80	single	125.38%	-
B05F.C030	B	Chiou-14-030	1.24%	1,000.00	160.00	285.00	38.16	single	14.31%	-
B06F.C050	B	Chiou-14-050	2.40%	1,000.00	160.00	198.10	13.28	single	10.81%	-
B07F.C065	B	Chiou-14-065	0.76%	1,000.00	160.00	285.00	22.96	single	25.44%	-
B08F.C069	B	Chiou-14-069	0.45%	1,000.00	160.00	211.20	15.92	single	17.88%	-
B09F.B066	B	BZ06-066	4.85%	1,000.00	160.00	285.00	39.20	single	38.72%	-
B10F.B148	B	BZ06-148	0.68%	1,000.00	160.00	292.10	9.92	single	9.07%	-
C01F.T001	C	SNPRC #13245	4.33%	1,000.00	6.92	40.00	20.80	single	65.00%	1.89
C02F.T002	C	SNPRC #14068	1.12%	1,000.00	1.80	40.00	6.80	single	6.41%	10.52
C03F.T005	C	SNPRC #25567	8.38%	1,000.00	13.40	40.00	29.48	single	170.96%	6.38
C04D.T002	C	SNPRC #14068	0.01%	8,000.00	1.00	40.00	5.24	serial	0.00%	24.46
C05F.T009	C	SNPRC #27958	17.40%	800.00	22.28	40.00	36.00	single	152.22%	13.29
C06F.T010	C	SNPRC #28064	4.40%	1,000.00	7.04	40.00	15.12	single	138.62%	19.02
C07F.C050	C	Chiou-14-050	2.99%	1,000.00	4.78	40.00	5.24	single	14.05%	7.92
C08D.C050	C	Chiou-14-050	0.82%	600.00	1.00	40.00	1.02	serial	0.00%	11.08
C09F.B051	C	BZ06-051	3.33%	500.00	2.66	40.00	2.92	single	49.45%	2.64
C10F.T005	C	SNPRC #25567	12.83%	1,000.00	1.50	40.00	14.08	single	221.02%	10.31
C11D.C069	C	Chiou-14-069	0.00%	1,000.00	1.00	40.00	0.76	serial	24.47%	6.81
C12D.B051	C	BZ06-051	0.36%	600.00	1.00	40.00	1.18	serial	14.76%	2.10
D01D.B220	D	BZ06-220	0.03%	1,000.00	1.00	40.00	0.70	serial	-	-
D02D.J001	D	BZ07-001	0.02%	1,000.00	1.00	40.00	2.39	serial	-	-
D03D.J007	D	BZ07-007	0.12%	947.20	1.00	40.00	0.95	serial	-	-
D04D.J029	D	BZ07-029	0.00%	1,000.00	1.00	40.00	2.25	serial	-	-
D05D.J032	D	BZ07-032	0.88%	1,000.00	1.00	40.00	0.97	serial	-	-
D06D.J034	D	BZ07-034	0.47%	1,000.00	1.00	40.00	2.23	serial	-	-
D07D.J039	D	BZ07-039	0.06%	1,000.00	1.00	40.00	1.05	serial	-	-
D08D.C057	D	Chiou-14-057	0.24%	1,000.00	1.00	40.00	0.90	serial	-	-
D09D.C003	D	Chiou-14-003	0.03%	1,000.00	1.00	40.00	0.88	serial	-	-
D10D.C044	D	Chiou-14-044	0.03%	913.96	1.00	40.00	0.93	serial	-	-
D11D.C041	D	Chiou-14-041	0.03%	1,000.00	1.00	40.00	1.31	serial	-	-
D12D.C042	D	Chiou-14-042	0.53%	1,000.00	1.00	40.00	0.78	serial	-	-
D13D.B221	D	BZ06-221	0.09%	548.96	1.00	40.00	0.66	serial	-	-
D14D.B227	D	BZ06-227	0.03%	1,000.00	1.00	40.00	0.52	serial	-	-
D15D.J004	D	BZ07-004	0.39%	1,000.00	1.00	40.00	0.69	serial	-	-
D16D.J005	D	BZ07-005	0.21%	1,000.00	1.00	40.00	0.46	serial	-	-
D17D.J030	D	BZ07-030	0.27%	988.80	1.00	40.00	0.68	serial	-	-
D18D.J035	D	BZ07-035	0.44%	1,000.00	1.00	40.00	0.70	serial	-	-
D19D.J041	D	BZ07-041	0.44%	1,000.00	1.00	40.00	0.53	serial	-	-
D20D.C001	D	Chiou-14-001	0.01%	1,000.00	1.00	40.00	0.64	serial	-	-
D21D.C004	D	Chiou-14-004	0.01%	1,000.00	1.00	40.00	0.53	serial	-	-
D22D.C065	D	Chiou-14-065	0.08%	1,000.00	1.00	40.00	0.48	serial	-	-

continued on next page

Supplemental Table S2 – continued from previous page

ID	Lib	Ind	PHB	TD	BV	TV	TY	NE	PHA	NDB
D23D.C030	D	Chiou-14-030	0.30%	629.26	1.00	40.00	0.63	serial	-	-
D24D.C039	D	Chiou-14-039	0.29%	1,000.00	1.00	40.00	0.57	serial	-	-
D25D.B218	D	BZ06-218	0.06%	893.52	1.00	40.00	< 0.40	serial	-	-
D26D.B224	D	BZ06-224	0.03%	795.70	1.00	40.00	< 0.40	serial	-	-
D27D.B225	D	BZ06-225	0.06%	1,000.00	1.00	40.00	< 0.40	serial	-	-
D28D.J042	D	BZ07-042	0.11%	1,000.00	1.00	40.00	< 0.40	serial	-	-
D29D.J045	D	BZ07-045	0.03%	985.60	1.00	40.00	< 0.40	serial	-	-
D30D.J047	D	BZ07-047	0.02%	1,000.00	1.00	40.00	< 0.40	serial	-	-
D31D.J100	D	BZ07-100	0.08%	1,000.00	1.00	40.00	0.42	serial	-	-
D32D.C036	D	Chiou-14-036	0.37%	154.76	1.00	40.00	0.42	serial	-	-
D33D.C054	D	Chiou-14-054	0.44%	1,000.00	1.00	40.00	< 0.40	serial	-	-
D34D.C056	D	Chiou-14-056	0.11%	1,000.00	1.00	40.00	< 0.40	serial	-	-
D35D.C058	D	Chiou-14-058	0.57%	627.80	1.00	40.00	< 0.40	serial	-	-
D36D.C059	D	Chiou-14-059	0.90%	383.98	1.00	40.00	< 0.40	serial	-	-
D37D.H003	D	Chiou-15-003	0.10%	220.46	1.00	40.00	< 0.40	serial	-	-
D38D.H004	D	Chiou-15-004	3.11%	186.88	1.00	40.00	< 0.40	serial	-	-
D39D.H005	D	Chiou-15-005	0.11%	511.00	1.00	40.00	< 0.40	serial	-	-
D40D.C005	D	Chiou-14-005	2.41%	182.50	1.00	40.00	< 0.40	serial	-	-

Supplemental Table S3: Library preparation and sequence mapping results. Key: ID, experiment ID (see Supplemental Table S2); Lib, library ID; T, tissue type; PHB, percent host DNA before; TD, total DNA used (ng); PID, pool ID; PC, total number of PCR amplification cycles; TR, total number of sequencing reads; RM, number of reads mapping to the baboon reference genome (papAnu2); PRM, percentage of reads mapping to the baboon reference genome (papAnu2).

ID	Lib	T	PHB	TD	PID	PC	TR	RM	PRM
A01F.T002	A	feces	0.1500%	83.00	A1	24	264,158	9,856	3.73%
A02F.T005	A	feces	4.4000%	200.00	A1	24	2,607,006	1,892,463	72.59%
A03F.T007	A	feces	0.0000%	191.00	A1	24	2,040,002	15,224	0.75%
A04F.T009	A	feces	13.5900%	200.00	A2	24	4,104,470	3,334,314	81.24%
A05F.B051	A	feces	1.5900%	122.00	A2	24	916,680	257,714	28.11%
A06F.B053	A	feces	1.1400%	120.00	A2	24	591,626	146,285	24.73%
A07B.T002	A	blood	-	200.00	A3	24	2,683,338	2,384,501	88.86%
A08B.T005	A	blood	-	200.00	A3	24	745,504	681,730	91.45%
A09B.T007	A	blood	-	200.00	A4	24	2,128,974	1,886,608	88.62%
A10B.T009	A	blood	-	200.00	A4	24	1,189,390	1,087,777	91.46%
B01F.T001	B	feces	3.5500%	60.06	B1	24	4,882,156	1,652,610	33.85%
B02F.T002	B	feces	0.1500%	13.27	B1	24	2,507,424	111,206	4.44%
B03F.T007	B	feces	0.0000%	22.11	B1	24	2,461,390	27,185	1.10%
B04F.T010	B	feces	9.8700%	65.01	B1	24	5,444,582	3,184,008	58.48%
B05F.C030	B	feces	1.2400%	33.39	B1	24	619,862	34,761	5.61%
B06F.C050	B	feces	2.4000%	11.62	B1	24	2,123,294	584,719	27.54%
B07F.C065	B	feces	0.7600%	20.09	B1	24	1,241,912	44,732	3.60%
B08F.C069	B	feces	0.4500%	13.93	B1	24	1,203,334	23,642	1.96%
B09F.B066	B	feces	4.8500%	34.30	B1	24	1,563,428	581,005	37.16%
B10F.B148	B	feces	0.6800%	8.68	B1	24	501,188	62,126	12.40%
B11B.T001	B	blood	-	196.50	B2	24	2,216,126	1,819,922	82.12%
B12B.T010	B	blood	-	201.50	B2	24	2,092,674	1,780,702	85.09%
C01F.T001	C	feces	4.3300%	17.68	C1	20	1,782,002	1,188,499	66.69%
C02F.T002	C	feces	1.1200%	5.78	C2	24	1,290,578	333,245	25.82%
C03F.T005	C	feces	8.3800%	25.06	C1	20	1,871,868	1,316,267	70.32%
C04D.T002	C	feces	0.0100%	3.41	C3	26	1,841,762	163,819	8.89%
C05F.T009	C	feces	17.4000%	30.60	C1	20	3,116,288	2,345,065	75.25%
C06F.T010	C	feces	4.4000%	12.85	C1	20	1,469,246	967,950	65.88%
C07F.C050	C	feces	2.9900%	4.45	C2	24	3,570,776	1,845,128	51.67%
C08D.C050	C	feces	0.8200%	0.67	C4	26	2,059,728	737,230	35.79%
C09F.B051	C	feces	3.3300%	2.48	C2	24	1,816,378	918,754	50.58%
C10F.T005	C	feces	12.8300%	11.97	C1	20	2,068,478	1,475,254	71.32%
C11D.C069	C	feces	0.0049%	0.49	C5	26	1,514,706	195,365	12.90%
C12D.B051	C	feces	0.3600%	0.77	C6	26	1,896,580	1,035,140	54.58%
D01D.B220	D	feces	0.0289%	0.53	D1	22	5,875,038	558,679	9.51%
D02D.J001	D	feces	0.0158%	1.79	D1	22	1,449,446	47,799	3.30%
D03D.J007	D	feces	0.1220%	0.71	D1	22	3,243,182	760,274	23.44%
D04D.J029	D	feces	0.0030%	1.69	D1	22	1,542,546	22,534	1.46%
D05D.J032	D	feces	0.8793%	0.73	D1	22	9,398,314	6,856,889	72.96%
D06D.J034	D	feces	0.4713%	1.67	D1	22	2,656,920	1,313,120	49.42%
D07D.J039	D	feces	0.0629%	0.79	D1	22	2,230,514	609,824	27.34%
D08D.C057	D	feces	0.2374%	0.67	D1	22	24,351,758	9,032,717	37.09%
D09D.C003	D	feces	0.0339%	0.66	D1	22	4,453,940	140,853	3.16%
D10D.C044	D	feces	0.0292%	0.70	D1	22	2,137,704	124,844	5.84%
D11D.C041	D	feces	0.0271%	0.98	D1	22	1,140,122	96,922	8.50%
D12D.C042	D	feces	0.5310%	0.59	D1	22	10,202,338	4,952,523	48.54%
D13D.B221	D	feces	0.0894%	0.49	D2	22	5,147,652	982,161	19.08%
D14D.B227	D	feces	0.0309%	0.39	D2	22	1,828,496	188,056	10.28%
D15D.J004	D	feces	0.3858%	0.52	D2	22	3,606,424	1,619,089	44.89%

continued on next page

Supplemental Table S3 – continued from previous page

ID	Lib	T	PHB	TD	PID	PC	TR	RM	PRM
D16D.J005	D	feces	0.2120%	0.35	D2	22	4,681,458	1,595,305	34.08%
D17D.J030	D	feces	0.2748%	0.51	D2	22	9,260,376	4,298,954	46.42%
D18D.J035	D	feces	0.4370%	0.52	D2	22	14,641,030	7,429,804	50.75%
D19D.J041	D	feces	0.4441%	0.40	D2	22	2,734,646	1,830,636	66.94%
D20D.C001	D	feces	0.0079%	0.48	D2	22	3,011,748	95,667	3.18%
D21D.C004	D	feces	0.0088%	0.40	D2	22	2,685,536	97,831	3.64%
D22D.C065	D	feces	0.0770%	0.36	D2	22	6,516,948	954,911	14.65%
D23D.C030	D	feces	0.2962%	0.47	D2	22	4,200,672	1,854,549	44.15%
D24D.C039	D	feces	0.2900%	0.43	D2	22	15,175,272	5,263,567	34.69%
D25D.B218	D	feces	0.0581%	0.23	D3	26	2,624,536	311,121	11.85%
D26D.B224	D	feces	0.0307%	0.23	D3	26	3,581,376	246,941	6.90%
D27D.B225	D	feces	0.0573%	0.23	D3	26	16,512,734	1,228,293	7.44%
D28D.J042	D	feces	0.1062%	0.23	D3	26	6,967,098	1,135,899	16.30%
D29D.J045	D	feces	0.0262%	0.23	D3	26	2,307,634	124,309	5.39%
D30D.J047	D	feces	0.0213%	0.23	D3	26	5,640,310	519,703	9.21%
D31D.J100	D	feces	0.0793%	0.31	D3	26	1,219,418	83,022	6.81%
D32D.C036	D	feces	0.3708%	0.32	D3	26	7,119,672	4,402,290	61.83%
D33D.C054	D	feces	0.4415%	0.23	D4	22	7,013,280	4,484,858	63.95%
D34D.C056	D	feces	0.1098%	0.23	D4	22	7,949,918	2,748,447	34.57%
D35D.C058	D	feces	0.5733%	0.23	D4	22	10,539,604	4,223,730	40.07%
D36D.C059	D	feces	0.8954%	0.23	D4	22	5,962,728	3,090,413	51.83%
D37D.H003	D	feces	0.1017%	0.23	D4	22	1,418,168	74,131	5.23%
D38D.H004	D	feces	3.1094%	0.23	D4	22	9,161,532	6,739,523	73.56%
D39D.H005	D	feces	0.1113%	0.23	D4	22	1,784,298	585,250	32.80%
D40D.C005	D	feces	2.4120%	0.23	D4	22	2,621,020	1,611,725	61.49%
E01B.T001	E	blood	-	200.00	E1	12	2,326,792	2,027,296	87.13%
E02B.T002	E	blood	-	200.00	E1	12	1,249,950	1,095,499	87.64%
E03B.T005	E	blood	-	200.00	E2	12	4,812,938	4,192,408	87.11%
E04B.T009	E	blood	-	200.00	E1	12	1,986,292	1,746,868	87.95%
E05B.T010	E	blood	-	200.00	E2	12	4,091,500	3,597,699	87.93%

Supplemental Table S4: DNA samples used for controlled experiments. Artificial "fecal" DNA was prepared by manually mixing DNA samples in controlled proportions. Artificial methylated DNA was also prepared using amplicons of lambda phage DNA (with known sequence) and methyl-transferase enzymes with specific recognition sites. 5,012 bp amplicons were prepared using the primers `/5Biosg/GTTCTGCACTGACAGATTAAAACTCG` and `CTGCTCATTAATATACTTCTGGGTTCC`, 15,089 bp amplicons were prepared using the primers `/5Biosg/GAGTGAATATATCGAACAGTCAGG` and `GTGTCATATTTCACTTCCGTACC`, and 10,144 bp amplicons were prepared using the primers `/5BiosG/ATAAAGATGAGACGCTGGAGTACA` and `GCGATAACCAGGTAAAATTTTCCG`. Key: ID, prepared DNA sample ID; DNA1, input DNA sample 1; DNA2, input DNA sample 2; PH, percentage of "host" (baboon) DNA; PB, percentage of bacterial DNA; L, length of DNA amplicon; Enz, methyltransferase enzyme(s) used; MD, CpG methylation density.

ID	DNA1	DNA2	PH	PB	L	Enz	MD
PB01	K12 *E. coli*	none	0.0%	100.00%	-	-	-
PB02	ATCC 11303 *E. coli*	none	0.0%	100.00%	-	-	-
PH01	Baboon blood	none	100.0%	0.0%	-	-	-
PH02	Baboon liver	none	100.0%	0.0%	-	-	-
AF01	Baboon blood	K12 *E. coli*	2.0%	98.0%	-	-	-
AF02	Baboon blood	K12 *E. coli*	0.2%	99.8%	-	-	-
AF03	Baboon blood	K12 *E. coli*	50.0%	50.0%	-	-	-
AF04	Baboon blood	K12 *E. coli*	2.0%	98.0%	-	-	-
AF05	Baboon blood	K12 *E. coli*	50.0%	50.0%	-	-	-
AF06	Baboon blood	K12 *E. coli*	5.0%	95.0%	-	-	-
AF07	Baboon blood	K12 *E. coli*	10.0%	90.0%	-	-	-
AF08	Baboon blood	ATCC 11303 *E. coli*	2.0%	98.0%	-	-	-
AF09	Baboon liver	ATCC 11303 *E. coli*	2.0%	98.0%	-	-	-
AF10	Baboon liver	ATCC 11303 *E. coli*	50.0%	50.0%	-	-	-
AF11	Baboon liver	ATCC 11303 *E. coli*	0.5%	99.5%	-	-	-
AF12	Baboon liver	ATCC 11303 *E. coli*	2.0%	98.0%	-	-	-
AF13	Baboon liver	ATCC 11303 *E. coli*	5.0%	95.0%	-	-	-
AF14	Baboon liver	ATCC 11303 *E. coli*	2.0%	98.0%	-	-	-
AF15	Baboon liver	ATCC 11303 *E. coli*	0.5%	99.5%	-	-	-
AF16	Baboon liver	ATCC 11303 *E. coli*	2.0%	98.0%	-	-	-
AF17	Baboon liver	ATCC 11303 *E. coli*	5.0%	95.0%	-	-	-
AF18	Baboon liver	ATCC 11303 *E. coli*	0.5%	99.5%	-	-	-
CD01	Lambda cl857 phage	none	0.0%	0.0%	5,012	*Hha*I	3.6
CD02	Lambda cl857 phage	none	0.0%	0.0%	5,012	-	0.0
CD03	Lambda cl857 phage	none	0.0%	0.0%	5,012	*Hha*I	3.6
CD04	Lambda cl857 phage	none	0.0%	0.0%	5,012	*Hha*I + *Hpa*II	7.2
CD05	Lambda cl857 phage	none	0.0%	0.0%	5,012	*Hha*I	3.6
CD06	Lambda cl857 phage	none	0.0%	0.0%	15,089	*Hha*I	6.9
CD07	Lambda cl857 phage	none	0.0%	0.0%	10,144	*Hha*I	6.3
CD08	Lambda cl857 phage	none	0.0%	0.0%	10,144	*Hha*I + *Hpa*II	17.7

Supplemental Table S5: Controlled DNA enrichment experiments. DNA enrichment was simulated from artificial "fecal" samples. In some cases, additional DNA was included. A number of variables described in Supplemental Protocol were tuned to evaluate their impact on enrichment results (see Supplemental Table S6). Key: ID, experiment ID; SID, experiment set ID; DNA1, input DNA sample 1 (see Supplemental Table S4 or this table); TD1, total amount of sample 1 (ng); PH, percentage of "host" (baboon) DNA in sample 1; DNA2, input DNA sample 2 (see Supplemental Table S4); TD2, total amount of sample 2 (ng); BV, volume of protein A beads used (μl); PV, volume of MBD-Fc protein used (μl); NC, NaCl concentration of reaction (μM); TV, total volume of reaction (μl); NW, number of washes; NCW, NaCl concentration of each wash (μM); WV, volume of each wash (μl); EM, Elution method. For elutions in TE, proteinase K was added at a ratio of 1 μl proteinase K to 10 μl 1X TE.

ID	SID	DNA1	TD1	PH	DNA2	TD2	BV	PV	NC	TV	NW	NCW	WV	EM
X001	S01	AF01	1,000.00	2.0%	-	0.00	160.0	16.00	150	166.20	0	-	-	150 μl TE
X002	S01	AF01	2,000.00	2.0%	-	0.00	160.0	16.00	150	172.40	0	-	-	150 μl TE
X003	S01	AF02	1,000.00	0.2%	-	0.00	160.0	16.00	150	164.00	0	-	-	150 μl TE
X004	S01	AF01	1,000.00	2.0%	-	0.00	320.0	32.00	150	326.20	0	-	-	150 μl TE
X005	S01	AF01	1,000.00	2.0%	-	0.00	80.0	8.00	150	86.20	0	-	-	150 μl TE
X006	S01	AF01	1,000.00	2.0%	-	0.00	40.0	4.00	150	46.20	0	-	-	150 μl TE
X007	S02	PB01	1,000.00	0.0%	-	0.00	160.0	16.00	150	163.60	0	-	-	150 μl TE
X008	S02	AF01	1,000.00	2.0%	-	0.00	16.0	1.60	150	22.20	0	-	-	150 μl TE
X009	S02	AF03	40.00	50.0%	-	0.00	80.0	8.00	150	130.00	0	-	-	150 μl TE
X010	S02	AF03	40.00	50.0%	-	0.00	40.0	4.00	150	90.00	0	-	-	150 μl TE
X011	S02	AF03	40.00	50.0%	-	0.00	16.0	1.60	150	66.00	0	-	-	150 μl TE
X012	S02	AF03	40.00	50.0%	-	0.00	8.0	0.80	150	58.00	0	-	-	150 μl TE
X013	S03	PB01	1,000.00	0.0%	-	0.00	160.0	0.00	150	163.60	0	-	-	150 μl TE
X014	S03	PB01	1,000.00	0.0%	-	0.00	40.0	0.00	150	47.20	0	-	-	150 μl TE
X015	S03	PB01	200.00	0.0%	-	0.00	40.0	0.00	150	47.20	0	-	-	150 μl TE
X016	S03	PB01	1,000.00	0.0%	-	0.00	40.0	32.00	150	47.20	0	-	-	150 μl TE
X017	S04	AF04	1,000.00	2.0%	-	0.00	1.0	0.10	150	7.20	0	-	-	150 μl TE
X018	S04	PB01	1,000.00	0.0%	-	0.00	40.0	4.00	150	43.60	0	-	-	150 μl TE
X019	S04	PB01	1,000.00	0.0%	-	0.00	40.0	4.00	300	58.20	0	-	-	150 μl TE
X020	S04	AF05	1,000.00	50.0%	-	0.00	40.0	4.00	150	105.80	0	-	-	150 μl TE
X021	S05	AF04	1,000.00	2.0%	-	0.00	40.0	4.00	150	46.20	0	-	-	150 μl TE
X022	S05	AF04	1,000.00	2.0%	-	0.00	40.0	4.00	150	46.20	0	-	-	150 μl TE
X023	S05	X001	29.16	51.0%	-	0.00	40.0	4.00	150	78.60	0	-	-	150 μl TE
X024	S05	X006	21.48	44.1%	-	0.00	40.0	4.00	150	78.40	0	-	-	150 μl TE
X025	S06	AF04	1,000.00	2.0%	CD01	500.00	40.0	4.00	150	48.90	0	-	-	150 μl TE
X026	S06	AF06	1,000.00	5.0%	-	0.00	40.0	4.00	150	49.90	0	-	-	150 μl TE
X027	S06	AF07	1,000.00	10.0%	-	0.00	40.0	4.00	150	56.10	0	-	-	150 μl TE
X028	S06	AF04	1,000.00	2.0%	CD02	500.00	40.0	4.00	150	48.70	0	-	-	150 μl TE
X029	S07	AF04	1,000.00	2.0%	CD01	2,500.00	40.0	4.00	150	58.70	0	-	-	150 μl TE
X030	S07	AF04	1,000.00	2.0%	-	0.00	1.0	0.10	150	7.20	0	-	-	150 μl TE
X031	S07	AF04	1,000.00	2.0%	-	0.00	1.0	0.10	150	100.00	0	-	-	150 μl TE
X032	S07	AF04	1,000.00	2.0%	CD01	500.00	1.0	0.10	150	9.70	0	-	-	150 μl TE
X033	S08	AF08	1,000.00	2.0%	-	0.00	8.0	0.80	150	13.00	0	-	-	150 μl TE
X034	S08	AF08	1,000.00	2.0%	-	0.00	4.0	0.40	150	9.00	0	-	-	150 μl TE
X035	S08	AF08	1,000.00	2.0%	-	0.00	2.0	0.20	150	7.00	0	-	-	150 μl TE
X036	S08	AF08	1,000.00	2.0%	-	0.00	0.5	0.05	150	6.00	0	-	-	150 μl TE
X037	S08	PB02	980.00	0.0%	-	0.00	1.0	0.10	150	7.20	0	-	-	150 μl TE
X038	S08	PH01	20.00	100.0%	-	0.00	1.0	0.10	150	7.20	0	-	-	150 μl TE
X039	S09	AF08	1,000.00	2.0%	CD03	1,000.00	40.0	4.00	150	56.30	0	-	-	150 μl TE
X040	S09	AF08	1,000.00	2.0%	CD03	500.00	40.0	4.00	150	50.60	0	-	-	150 μl TE
X041	S10	AF09	1,000.00	2.0%	CD04	500.00	40.0	4.00	150	80.00	0	-	-	150 μl TE
X042	S10	AF09	1,000.00	2.0%	CD04	500.00	16.0	1.60	150	32.00	0	-	-	150 μl TE

continued on next page

Supplemental Table S5 – continued from previous page

ID	EID	DNA1	TD1	PH	DNA2	TD2	BV	PV	NC	TV	NW	NCW	WV	EM
X043	S11	PH02	1,000.00	100.0%	-	0.00	1.0	0.10	150	12.13	0	-	-	150 µl TE
X044	S11	PH02	2,000.00	100.0%	-	0.00	1.0	0.10	150	23.36	0	-	-	150 µl TE
X045	S11	PH02	1,000.00	100.0%	-	0.00	2.0	0.20	150	13.13	0	-	-	150 µl TE
X046	S11	PH02	1,000.00	100.0%	-	0.00	4.0	0.40	150	15.13	0	-	-	150 µl TE
X047	S11	PH02	1,000.00	100.0%	-	0.00	8.0	0.80	150	19.13	0	-	-	150 µl TE
X048	S11	PH02	1,000.00	100.0%	-	0.00	16.0	1.60	150	27.13	0	-	-	150 µl TE
X049	S12	PH02	112.00	100.0%	-	0.00	1.0	0.10	150	2.25	0	-	-	150 µl TE
X050	S12	PH02	112.00	100.0%	-	0.00	1.0	0.10	150	2.25	0	-	-	40 µl TE
X051	S12	PH02	112.00	100.0%	-	0.00	1.0	0.10	150	2.25	0	-	-	150 µl TE
X052	S12	PH02	112.00	100.0%	-	0.00	1.0	0.10	150	2.25	0	-	-	60 µl TE
X053	S13	PB02	1,000.00	0.0%	CD05	1,000.00	40.0	4.00	150	80.00	0	-	-	150 µl TE
X054	S13	AF10	2,000.00	50.0%	-	0.00	40.0	4.00	150	80.00	0	-	-	150 µl TE
X055	S14	PB02	1,000.00	0.0%	CD06	1,000.00	40.0	4.00	150	80.00	0	-	-	150 µl TE
X056	S14	AF11	1,000.00	0.5%	CD06	1,000.00	40.0	4.00	150	80.00	0	-	-	150 µl TE
X057	S14	AF12	1,000.00	2.0%	CD06	1,000.00	40.0	4.00	150	80.00	0	-	-	150 µl TE
X058	S14	AF13	1,000.00	5.0%	CD06	1,000.00	40.0	4.00	150	80.00	0	-	-	150 µl TE
X059	S15	PB02	1,000.00	0.0%	-	0.00	40.0	4.00	150	80.00	1	150	80	see Supplemental Table S7
X060	S15	PH02	250.00	100.0%	-	0.00	40.0	4.00	150	80.00	1	150	80	see Supplemental Table S7
X061	S16	AF14	1,000.00	2.0%	-	0.00	40.0	4.00	150	80.00	0	-	-	2 M NaCl
X062	S16	AF14	1,000.00	2.0%	-	0.00	40.0	1.00	150	80.00	0	-	-	2 M NaCl
X063	S16	AF14	1,000.00	2.0%	CD07	1,000.00	40.0	4.00	150	80.00	0	-	-	2 M NaCl
X064	S16	AF14	1,000.00	2.0%	CD07	1,000.00	40.0	4.00	150	80.00	0	-	-	2 M NaCl
X065	S16	AF14	1,000.00	2.0%	-	0.00	40.0	4.00	200	80.00	0	-	-	2 M NaCl
X066	S16	AF14	1,000.00	2.0%	-	0.00	40.0	4.00	150	80.00	1	200	200	2 M NaCl
X067	S17	AF14	1,000.00	2.0%	-	0.00	40.0	4.00	150	80.00	0	-	-	150 µl TE
X068	S17	AF14	1,000.00	2.0%	-	0.00	40.0	4.00	150	80.00	0	-	-	2 M NaCl
X069	S17	AF14	1,000.00	2.0%	-	0.00	40.0	0.25	150	80.00	0	-	-	2 M NaCl
X070	S17	AF14	1,000.00	2.0%	-	0.00	40.0	0.25	150	80.00	1	150	100	2 M NaCl
X071	S17	AF14	1,000.00	2.0%	-	0.00	40.0	0.25	150	80.00	1	200	100	2 M NaCl
X072	S17	AF14	1,000.00	2.0%	CD07	1,000.00	40.0	0.25	150	80.00	1	200	100	2 M NaCl
X073	S18	AF14	1,000.00	2.0%	-	0.00	40.0	1.00	150	80.00	1	150	100	2 M NaCl
X074	S18	AF14	1,000.00	2.0%	-	0.00	40.0	1.00	150	80.00	1	200	100	2 M NaCl
X075	S18	AF14	2,000.00	2.0%	-	0.00	40.0	1.00	150	80.00	1	150	100	2 M NaCl
X076	S19	AF15	1,000.00	0.5%	-	0.00	40.0	1.00	150	80.00	1	150	100	2 M NaCl
X077	S19	AF15	1,000.00	0.5%	-	0.00	40.0	1.00	150	80.00	2	150	100	2 M NaCl
X078	S19	AF15	1,000.00	0.5%	-	0.00	40.0	1.00	150	80.00	3	150	100	2 M NaCl
X079	S20	AF15	1,000.00	0.5%	-	0.00	40.0	1.00	150	80.00	1	150	80	2 M NaCl
X080	S20	AF15	1,000.00	0.5%	-	0.00	40.0	1.00	150	80.00	1	150	40	2 M NaCl
X081	S20	AF15	1,000.00	0.5%	CD07	40.00	40.0	1.00	150	80.00	1	150	80	2 M NaCl
X082	S20	PB02	1,000.00	0.0%	CD07	40.00	40.0	1.00	150	80.00	1	150	80	2 M NaCl
X083	S21	AF14	1,000.00	2.0%	-	0.00	40.0	1.00	150	80.00	1	150	80	2 M NaCl
X084	S21	AF14	1,000.00	2.0%	-	0.00	40.0	1.00	150	80.00	1	150	200	2 M NaCl
X085	S21	AF14	1,000.00	2.0%	-	0.00	40.0	4.00	150	80.00	1	150	80	2 M NaCl
X086	S21	AF15	1,000.00	0.5%	-	0.00	40.0	4.00	150	80.00	1	150	80	2 M NaCl
X087	S21	AF15	1,000.00	0.5%	CD08	20.00	40.0	4.00	150	80.00	1	150	80	2 M NaCl
X088	S21	PB02	1,000.00	0.0%	CD08	20.00	40.0	4.00	150	80.00	1	150	80	2 M NaCl
X089	S22	AF15	1,000.00	0.5%	-	0.00	40.0	1.00	150	80.00	1	150	80	2 M NaCl
X090	S22	AF15	1,000.00	0.5%	CD08	20.00	40.0	1.00	150	80.00	1	150	80	2 M NaCl
X091	S22	AF14	1,000.00	2.0%	CD08	20.00	40.0	1.00	150	80.00	1	150	80	2 M NaCl
X092	S22	PB02	1,000.00	0.0%	CD08	20.00	40.0	1.00	150	80.00	1	150	80	2 M NaCl
X093	S23	AF15	1,000.00	0.5%	-	0.00	40.0	0.25	150	80.00	1	150	100	2 M NaCl
X094	S23	AF15	1,000.00	0.5%	-	0.00	40.0	0.25	150	80.00	2	150	100	2 M NaCl
X095	S23	AF15	1,000.00	0.5%	-	0.00	40.0	0.25	150	80.00	3	150	100	2 M NaCl
X096	S23	AF15	1,000.00	0.5%	-	0.00	40.0	0.25	150	80.00	4	150	100	2 M NaCl
X097	S24	AF15	1,000.00	0.5%	-	0.00	40.0	0.25	150	80.00	1	150	100	2 M NaCl

continued on next page

Supplemental Table S5 – continued from previous page

ID	EID	DNA1	TD1	PH	DNA2	TD2	BV	PV	NC	TV	NW	NCW	WV	EM
X098	S24	AF15	1,000.00	0.5%	-	0.00	2.5	0.25	150	80.00	1	150	100	2 M NaCl
X099	S24	AF16	1,000.00	2.0%	-	0.00	40.0	0.25	150	80.00	1	150	100	2 M NaCl
X100	S24	AF17	1,000.00	5.0%	-	0.00	40.0	0.25	150	80.00	1	150	100	2 M NaCl
X101	S25	AF15	1,000.00	0.5%	-	0.00	2.5	0.25	150	40.00	1	150	100	2 M NaCl
X102	S25	AF16	1,000.00	2.0%	-	0.00	10.0	1.00	150	40.00	1	150	100	2 M NaCl
X103	S25	AF17	1,000.00	5.0%	-	0.00	25.0	2.50	150	40.00	1	150	100	2 M NaCl
X104	S25	AF15	1,000.00	0.5%	-	0.00	2.5	0.25	150	40.00	1	150	40	2 M NaCl
X105	S25	AF16	1,000.00	2.0%	-	0.00	3.2	0.32	150	40.00	1	150	100	2 M NaCl
X106	S25	AF15	1,000.00	0.5%	-	0.00	0.8	0.08	150	40.00	1	150	100	2 M NaCl
X107	S26	AF15	1,000.00	0.5%	-	0.00	2.5	0.25	150	40.00	1	150	100	2 M NaCl
X108	S26	AF15	1,000.00	0.5%	-	0.00	0.8	0.08	150	40.00	1	150	100	2 M NaCl
X109	S26	AF15	1,000.00	0.5%	-	0.00	2.5	0.25	150	40.00	1	150	100	2 M NaCl
X110	S27	AF17	1,000.00	0.5%	-	0.00	0.8	0.08	150	40.00	1	150	100	2 M NaCl
X111	S27	AF17	1,000.00	0.5%	-	0.00	0.8	0.08	150	40.00	1	200	100	2 M NaCl
X112	S27	AF17	1,000.00	0.5%	-	0.00	0.8	0.08	150	40.00	1	350	100	2 M NaCl
X113	S27	AF17	1,000.00	0.5%	-	0.00	0.8	0.08	150	40.00	1	450	100	2 M NaCl
X114	S27	AF17	4,000.00	0.5%	-	0.00	3.2	0.32	150	40.00	1	150	100	2 M NaCl
X115	S27	AF16	1,000.00	2.0%	-	0.00	3.2	0.32	150	40.00	1	150	100	2 M NaCl
X116	S28	AF18	1,000.00	0.5%	-	0.00	0.8	0.08	150	40.00	1	150	100	2 M NaCl
X117	S28	AF18	1,000.00	0.5%	-	0.00	0.8	0.08	150	40.00	1	200	100	2 M NaCl
X118	S28	AF18	1,000.00	0.5%	-	0.00	0.8	0.08	150	40.00	1	350	100	2 M NaCl
X119	S28	AF18	1,000.00	0.5%	-	0.00	0.8	0.08	150	40.00	1	450	100	2 M NaCl
X120	S28	X107	2.40	27.0%	-	0.00	1.3	0.13	150	40.00	1	150	100	2 M NaCl

Supplemental Table S6: Controlled DNA enrichment experiment results. Percentages of host and bacterial DNA before and after enrichment experiments listed in Supplemental Table S5 were estimated by qPCR using host- and bacteria-specific primers (see Supplemental Protocol). Key: ID, experiment ID (see Supplemental Table S5); PHB, percentage of host DNA before; PHA, percentage of host DNA after; PBA, percentage of bacterial DNA after; TY, total DNA yield (ng); HY, estimated host DNA yield (ng); BY, estimated bacterial DNA yield (ng).

ID	PHB	PHA	PBA	TY	HY	BY
X001	2.0%	50.98%	56.74%	38.88	19.82	22.06
X002	2.0%	42.23%	49.32%	73.60	31.08	36.30
X003	0.2%	5.10%	101.77%	20.32	1.04	20.68
X004	2.0%	45.10%	62.70%	39.20	17.68	24.58
X005	2.0%	60.54%	46.75%	32.64	19.76	15.26
X006	2.0%	44.13%	45.74%	28.64	12.64	13.10
X007	0.0%	2.72%	110.93%	38.24	1.04	42.42
X008	2.0%	51.49%	51.28%	28.20	14.52	14.46
X009	50.0%	120.19%	6.43%	17.24	20.72	1.11
X010	50.0%	132.00%	3.18%	19.00	25.08	0.60
X011	50.0%	154.86%	1.86%	11.52	17.84	0.21
X012	50.0%	140.93%	1.76%	14.12	19.90	0.25
X013	0.0%	-	-	4.28	-	-
X014	0.0%	-	-	5.28	-	-
X015	0.0%	-	-	2.84	-	-
X016	0.0%	-	-	44.80	-	-
X017	2.0%	60.02%	3.18%	16.36	9.82	0.52
X018	0.0%	-	-	18.56	-	-
X019	0.0%	-	-	9.68	-	-
X020	50.0%	86.25%	0.48%	464.00	400.20	2.23
X021	2.0%	58.69%	29.84%	24.40	14.32	7.28
X022	2.0%	41.37%	56.03%	23.88	9.88	13.38
X023	51.0%	29.76%	15.36%	0.50	0.15	0.08
X024	44.1%	2.19%	8.89%	0.56	0.01	0.05
X025	2.0%	11.68%	10.47%	114.00	13.32	11.94
X026	5.0%	70.00%	18.42%	54.40	38.08	10.02
X027	10.0%	98.29%	9.19%	93.60	92.00	8.60
X028	2.0%	46.22%	37.09%	35.48	16.40	13.16
X029	2.0%	3.90%	3.26%	346.80	13.53	11.31
X030	2.0%	47.61%	4.07%	24.28	11.56	0.99
X031	2.0%	40.93%	11.19%	23.60	9.66	2.64
X032	2.0%	47.85%	1.96%	25.12	12.02	0.49
X033	2.0%	45.76%	41.10%	24.04	11.00	9.88
X034	2.0%	39.32%	28.48%	18.12	7.12	5.16
X035	2.0%	74.85%	13.87%	15.04	11.26	2.09
X036	2.0%	60.00%	16.84%	12.80	7.68	2.16
X037	0.0%	0.00%	73.67%	2.56	0.00	1.89
X038	100.0%	115.83%	0.01%	12.76	14.78	0.00
X039	2.0%	7.90%	6.71%	158.80	12.54	10.66
X040	2.0%	10.89%	10.28%	98.80	10.76	10.16
X041	2.0%	7.67%	9.32%	232.80	17.86	21.70
X042	2.0%	35.24%	38.99%	41.60	14.66	16.22
X043	100.0%	-	-	38.40	-	-
X044	100.0%	-	-	42.40	-	-
X045	100.0%	-	-	78.80	-	-
X046	100.0%	-	-	141.60	-	-
X047	100.0%	-	-	282.40	-	-

continued on next page

Supplemental Table S6 – continued from previous page

ID	PHB	PHA	PBA	TY	HY	BY
X048	100.0%	-	-	456.00	-	-
X049	100.0%	-	-	17.52	-	-
X050	100.0%	-	-	14.64	-	-
X051	100.0%	-	-	1.89	-	-
X052	100.0%	-	-	3.19	-	-
X053	0.0%	-	2.59%	347.20	-	9.00
X054	50.0%	-	2.19%	496.00	-	10.86
X055	0.0%	-	10.08%	206.40	-	20.80
X056	0.5%	0.88%	6.01%	220.40	1.95	13.24
X057	2.0%	5.32%	3.21%	204.40	10.88	6.56
X058	5.0%	12.17%	2.88%	222.40	27.06	6.40
X059	0.0%	-	-	-	-	-
X060	100.0%	-	-	-	-	-
X061	2.0%	60.57%	19.32%	18.36	11.12	3.55
X062	2.0%	83.19%	12.39%	14.40	11.98	1.78
X063	2.0%	2.10%	0.25%	680.00	14.28	1.70
X064	2.0%	1.97%	0.30%	656.00	12.92	1.97
X065	2.0%	58.82%	18.97%	16.32	9.60	3.10
X066	2.0%	101.43%	5.27%	9.76	9.90	0.51
X067	2.0%	55.65%	16.44%	16.28	9.06	2.68
X068	2.0%	65.89%	21.69%	17.24	11.36	3.74
X069	2.0%	78.68%	17.98%	12.76	10.04	2.29
X070	2.0%	128.57%	3.58%	6.44	8.28	0.23
X071	2.0%	114.15%	2.56%	6.36	7.26	0.16
X072	2.0%	28.33%	0.82%	26.12	7.40	0.21
X073	2.0%	144.68%	4.86%	8.64	12.50	0.42
X074	2.0%	133.61%	4.28%	7.20	9.62	0.31
X075	2.0%	137.91%	6.36%	15.88	21.90	1.01
X076	0.5%	237.32%	30.14%	2.84	6.74	0.86
X077	0.5%	357.50%	24.50%	2.40	8.58	0.59
X078	0.5%	270.16%	17.74%	2.48	6.70	0.44
X079	0.5%	234.81%	33.86%	3.16	7.42	1.07
X080	0.5%	257.07%	44.62%	3.68	9.46	1.64
X081	0.5%	20.03%	2.15%	29.16	5.84	0.63
X082	0.0%	0.00%	3.59%	26.76	0.00	0.96
X083	2.0%	240.00%	8.42%	9.00	21.60	0.76
X084	2.0%	243.69%	10.49%	8.24	20.08	0.86
X085	2.0%	233.55%	20.94%	9.36	21.86	1.96
X086	0.5%	227.66%	66.81%	3.76	8.56	2.51
X087	0.5%	43.31%	12.76%	16.44	7.12	2.10
X088	0.0%	0.00%	14.38%	14.28	0.00	2.05
X089	0.5%	224.26%	45.29%	2.72	6.10	1.23
X090	0.5%	46.20%	8.26%	16.32	7.54	1.35
X091	2.0%	68.14%	3.79%	19.40	13.22	0.74
X092	0.0%	0.00%	7.89%	15.56	0.00	1.23
X093	0.5%	86.44%	7.81%	2.64	2.28	0.21
X094	0.5%	73.31%	4.84%	2.36	1.73	0.11
X095	0.5%	106.31%	3.65%	1.74	1.85	0.06
X096	0.5%	114.75%	3.60%	1.42	1.63	0.05
X097	0.5%	127.97%	40.42%	2.36	3.02	0.95
X098	0.5%	130.90%	28.13%	2.88	3.77	0.81
X099	2.0%	106.34%	8.35%	13.56	14.42	1.13
X100	5.0%	135.67%	5.32%	17.44	23.66	0.93
X101	0.5%	49.11%	40.65%	2.48	1.22	1.01
X102	2.0%	83.82%	8.76%	13.60	11.40	1.19

continued on next page

Supplemental Table S6 – continued from previous page

ID	PHB	PHA	PBA	TY	HY	BY
X103	5.0%	96.18%	13.18%	20.40	19.62	2.69
X104	0.5%	91.02%	50.19%	2.16	1.97	1.08
X105	2.0%	74.20%	6.46%	12.56	9.32	0.81
X106	0.5%	-	-	-	-	-
X107	0.5%	139.38%	72.94%	3.20	4.46	2.33
X108	0.5%	101.79%	46.19%	2.68	2.73	1.24
X109	0.5%	124.12%	45.74%	2.72	3.38	1.24
X110	0.5%	98.30%	32.05%	2.24	2.20	0.72
X111	0.5%	138.57%	21.16%	1.96	2.72	0.41
X112	0.5%	128.51%	40.00%	1.48	1.90	0.59
X113	0.5%	137.04%	83.33%	1.08	1.48	0.90
X114	0.5%	113.78%	21.12%	7.84	8.92	1.66
X115	2.0%	141.15%	2.73%	10.40	14.68	0.28
X116	0.5%	60.37%	53.60%	2.72	1.64	1.46
X117	0.5%	92.22%	45.12%	2.42	2.23	1.09
X118	0.5%	68.13%	30.86%	1.78	1.21	0.55
X119	0.5%	77.34%	66.41%	2.05	1.59	1.36
X120	27.0%	170.44%	3.55%	0.55	0.94	0.02

Supplemental Table S7: Controlled DNA enrichment elution series. After hybridizing DNA to MBD-bound beads, bound DNA was eluted in a series with progressively higher NaCl concentrations. The quantity of DNA in each elution was then quantified by Qubit. Key: ID, experiment ID (see Supplemental Table S5); EN, elution number (elution 0 represents a wash); NC, NaCl concentration of reaction (μM); EV, Elution volume (μl); EY, elution DNA yield (ng); CY, cumulative DNA yield including previous elutions in the series (ng).

ID	E	NC	EV	EY	CY
X059	0	150	80	864.00	864.00
X059	1	200	80	27.04	891.04
X059	2	350	80	2.93	893.97
X059	3	450	80	1.17	895.14
X059	4	600	80	0.00	895.14
X059	5	1000	80	0.00	895.14
X059	6	2000	80	0.00	895.14
X060	0	150	80	62.56	62.56
X060	1	200	80	15.76	78.32
X060	2	350	80	49.44	127.76
X060	3	450	80	67.84	195.60
X060	4	600	80	100.80	296.40
X060	5	1000	80	34.24	330.64
X060	6	2000	80	2.26	332.90

Supplemental Protocol

FecalSeq enrichment protocol

Portions of this protocol are modified from the NEBNext Microbiome DNA Enrichment Kit manual (New England Biolabs cat. #E2612S)

Materials and reagents

- Extracted fecal-derived DNA of known quantity
- NEBNext Microbiome DNA Enrichment Kit (New England Biolabs; cat. #E2612S or #E2612L)
- Rotating mixer
- Magnetic rack for 1.5/2.0 ml microcentrifuge tubes
- 5 M NaCl

Before beginning

1. Extract and prepare DNA samples

   While any fecal DNA (fDNA) extraction method should in principle be compatible with the MBD enrichment, methods that maximize the recovery of host DNA are preferable. Bead-beating methods that increase total DNA yield from feces, for example, should be avoided because the mechanical disruption increases the yield of cell-wall-bound DNA (i.e., from bacteria or plants) while fragmenting host DNA.

   We suggest aiming for a total yield of 1 μg of DNA for all samples in a maximum volume of 30 μl each, although we have had success with as little as 500 ng (the yield of host DNA is likely more important than the yield of total fDNA). If the volume is greater than 30 μl, the DNA can be concentrated via a bead cleanup (Auxiliary protocol A).

   Prior to enrichment, DNA should be quantified for the total yield (e.g., by fluorometer or spectrophotometer). Ideally, the host DNA should also be quantified by qPCR (Auxiliary protocol B).

2. Calculate the required volume of MBD2-Fc-bound magnetic beads (hereafter referred to as "MBD beads") for each enrichment reaction, as well as the total volume for a set of reactions as follows.

   As an approximate rule, prepare 1 μl of MBD beads for every 6.25 ng of target host DNA in each enrichment reaction. If samples contain less than 6.25 ng of host DNA or if the amount of host DNA is not quantified, prepare 1 μl of MBD beads.

   We recommend preparing batches of MBD beads (see step 5) with a minimum volume of 40 μl, as lower volumes preclude adequate mixing. If a smaller volume is needed, leftover unused MBD beads can be stored at 4 °C for up to a week.

3. Resuspend protein A magnetic beads by gently pipetting the mixture up and down until the suspension is homogenous, or by slowly rotating the mixture at 4 °C for 15 minutes. **Do not vortex**.

4. Prepare 1X bind/wash buffer by diluting 1 part 5X bind/wash buffer with 4 parts DNase-free water. As a general rule, the volume of 1X bind/wash buffer needed can be calculated as:

   2.5 ml + 1.2 ml × [number of enrichment reactions]

   The amount of 1X bind/wash buffer depends on the total volume of MBD beads and the total number of enrichment reactions. MBD beads can be prepared with a maximum volume of 160 μl in a single reaction.

As very small volumes (1 – 8 μl) of beads are needed for our enrichment method, a single bead preparation reaction is nearly always sufficient. If more beads are needed, increase the number of bead preparation reactions and adjust the volume of 1X bind/wash buffer accordingly. Alternatively, for volumes up to 320 μl, prepare an additional 1 ml of 1X bind/wash buffer per bead preparation reaction and add an extra wash step (see step 14).

2.5 ml of 1X bind/wash buffer are required for a single bead preparation reaction up to 160 μl. Prepare an additional 1.2 ml of 1X bind/wash buffer per enrichment reaction. This number takes into account the volume needed to prepare 2 M NaCl elution buffer in the following step.

Keep 1X bind/wash buffer on ice throughout the MBD bead preparation. For the wash steps following the capture reaction, 1X bind/wash buffer can be at room temperature.

5. Prepare 2 M NaCl elution buffer by diluting 5 M NaCl with 1X bind/wash buffer. 100 μl of 2 M NaCl elution buffer are needed per enrichment reaction.

   1X bind/wash buffer has a NaCl concentration of 150 mM. 1 ml of 2 M NaCl elution buffer can be prepared by adding 370 μl of 5 M NaCl with 630 μl of 1X bind/wash buffer.

Preparing MBD beads

6. If preparing 40 μl of MBD beads, add 4 μl of MBD2-Fc protein to 40 μl of protein A magnetic beads in a 1.5 ml microcentrifuge tube. For preparing other volumes (*n* μl) of MBD beads, add *n*/10 μl MBD2-Fc protein to *n* μl of protein A magnetic beads.

   As a rule, we do not prepare less than 40 μl of MBD beads due to diminished efficiency of both rotational mixing and magnetic separation at low volumes.

7. Mix the bead-protein mixture by rotating the tube in a rotating mixer for 10 minutes at room temperature.
8. Briefly spin the tube and place on the magnetic rack for 2 – 5 minutes until the beads have collected to the wall of the tube and the solution is clear.
9. Carefully remove and discard the supernatant with a pipette without disturbing the beads.
10. Add 1 ml of 1X bind/wash buffer (kept on ice) to the tube to wash the beads. Pipette up and down a few times to mix.
11. Mix the beads by rotating the tube in a rotating mixer for 3 minutes at room temperature.
12. Briefly spin the tube and place on the magnetic rack for 2 – 5 minutes until the beads have collected to the wall of the tube and the solution is clear.
13. Carefully remove and discard the supernatant with a pipette without disturbing the beads.
14. Repeat steps 10 – 13.

    If preparing between 160 μl and 320 μl of beads, repeat steps 10 – 13 twice for a total of three washes to ensure the removal of unbound MBD2-Fc protein.

15. Remove the tube from the rack and add *n* μl (determined in step 6) of 1X bind/wash buffer to resuspend the beads. Mix by pipetting the mixture up and down until the suspension is homogenous.

Capture methylated host DNA

Since reaction volumes are well under 100 µl, multiple enrichment reactions can be processed together in a microplate, with pipetting steps conducted using a multichannel pipettor. Compatible rotating mixers and magnetic separators would also be required. Here, we proceed to describe the capture procedure using a 1.5 ml tube.

The total volume of the capture reaction is an important consideration. We have observed decreased DNA binding efficiency when the concentration of MBD beads or DNA in the capture reaction is low. We therefore recommend maintaining a total reaction volume of approximately 40 µl, as we have experienced consistent success with this volume even when adding as little as 1 µl of MBD beads. Decreasing the reaction volume may result in decreased efficacy of rotational mixing. It is a good idea to keep the volume of all reactions consistent as this facilitates processing of many samples and, if DNA amounts and bead volumes are kept consistent, serves as a control for the effects of bead or DNA concentration on enrichment efficiency. Our subsequent procedures assume a reaction volume of 40 µl (not including MBD beads). If using other reaction volumes, pay particular attention to notes following each step in this section.

16. Aliquot 8 µl of 5X bind/wash buffer to a 1.5 ml microcentrifuge tube

    For reaction volumes other than 40 µl, tune the volume of 5X bind/wash buffer to maintain 1X concentration and adjust accordingly the volume of DNase-free water added in step 17. The volume of MBD beads should be excluded from this calculation as prepared MBD beads are already at 1X concentration.

    We recommend equilibrating 5X bind/wash buffer to room temperature prior to aliquoting for more accurate pipetting.

17. Add up to 30 µl of DNA (prepared in step 1) to the tube. Bring the total volume to 40 µl with DNase-free water.

    For reaction volumes other than 40 µl, adjust the volume of DNase-free water added to reach the target volume. Be sure to maintain 1X bind/wash concentration.

18. Add MBD beads to the tube using the volume determined in step 2. Pipette the mixture up and down or swirl a few times to mix.

    As an approximate rule and as stated above, add 1 µl of MBD beads for every 6.25 ng of target host DNA in each enrichment reaction. If samples contain less than 6.25 ng of host DNA or if the amount of host DNA is not quantified, add 1 µl of MBD beads.

19. Incubate the reaction for 15 minutes at room temperature with rotation.

20. Following incubation at room temperature, briefly spin the tube and place on the magnetic rack for 5 minutes until the beads have collected to the wall and the solution is clear.

21. Carefully remove the supernatant with a pipette without disturbing the beads. The supernatant is enriched for microbial DNA and may be saved and purified by bead cleanup (Auxiliary protocol A). Otherwise, discard the supernatant.

22. Add 1 ml of 1 bind/wash buffer (kept at room temperature) to wash the beads.

    If processing in a microplate, decrease the volume of wash buffer to 100 µl.

23. Carefully remove and discard the wash buffer with a pipette without disturbing the beads.

24. *Optional*. Add 100 µl of 1X bind/wash buffer (kept at room temperature) to the beads. Pipette the mixture up and down a few times to mix.

    We have found that an additional wash with 100 µl of 1X bind/wash buffer followed by rotation (steps 24 – 27) substantially improved enrichment. To skip this wash, proceed to step 28.

25. Mix the beads by rotating the tube in a rotating mixer for 3 minutes at room temperature.
26. Briefly spin the tube and place on the magnetic rack for 2 – 5 minutes until the beads have collected to the wall of the tube and the solution is clear.
27. Carefully remove and discard the supernatant with a pipette without disturbing the beads.

Eluting captured host DNA

The NEBNext Microbiome Enrichment Kit includes an elution protocol for captured DNA that includes digestion of DNA-bound MBD beads with proteinase K and elution with TE buffer. We have found that elution with 2 M NaCl is just as effective, is less time consuming, and conserves proteinase K. Most importantly, we have found that DNA samples eluted with 2 M NaCl and purified by bead cleanup can be further enriched in a repeat enrichment reaction. DNA samples eluted with proteinase K and TE buffer and purified by bead cleanup in contrast produced miniscule yields following a repeat enrichment reaction.

28. Add 100 µl of 2 M NaCl (prepared in step 5 and kept at room temperature) to the beads. Pipette the mixture up and down a few times to mix.

    If large numbers of samples are being processed, considering lowering the elution volume such that the combined volume of DNA and SPRI beads (see Auxiliary protocol A; step 1) does not exceed the capacity of microplate wells and thereby preclude the ability to parallelize bead cleanups.

29. Mix the beads by rotating the tube in a rotating mixer for 3 minutes at room temperature.
30. Briefly spin the tube and place on the magnetic rack for 2 – 5 minutes until the beads have collected to the wall of the tube and the solution is clear.
31. Carefully remove the supernatant to a fresh microcentrifuge tube and discard beads.
32. Proceed to bead cleanup to purify sample (Auxiliary protocol A).

Auxiliary protocols

Auxiliary protocol A: Bead cleanup

Portions of this protocol are modified from Pacific Biosciences protocol # 001-252-177-03.

Materials and reagents

- Pre-washed magnetic SPRI beads, prepared following Rohland and Reich (2012)
- 70% ethanol, freshly prepared
- 1X TE buffer
- Magnetic stand

- Centrifuge

Procedures

1. Add 1.5X – 1.8x volume of pre-washed magnetic beads to DNA in a 1.5 ml tube.

   If the combined volume of beads and DNA does not exceed the capacity of the tube or well, large numbers of bead cleanups can be conducted in parallel on a microplate.

2. Mix the bead/DNA solution thoroughly by pipetting up and down several times.

3. Vortex the beads for 5 minutes.

4. Briefly spin the tube and place on the magnetic rack for 5 minutes or until the solution is clear.

5. Carefully remove and discard the supernatant without disturbing the beads.

6. Wash beads with freshly prepared 70% ethanol. Wait 1 minute, then pipette and discard the ethanol.

   Use a sufficient volume of 70% ethanol to completely cover the bead pellet (e.g., 100 µl for microplates and 400 µl for 1.5 ml tubes). Slowly dispense the 70% ethanol against the side of the tube opposite the beads. Do not disturb the bead pellet.

7. Repeat step 6 above.

8. Remove residual 70% ethanol and air-dry the bead pellet for 1 minute.

   Spin at full speed for 2 minutes in order to collect residual 70% ethanol. Then place on the magnetic rack for 30 seconds before pipetting the residual 70% ethanol and air-drying for 1 minute.

9. Resuspend the beads in 30 – 40 µl of 1X TE buffer or another suitable DNA stabilization buffer.

10. Vortex for 1 minute, then incubate for 2 minutes. Spin the sample at full speed to pellet beads. Return to the magnet and collect the supernatant in a new 1.5 ml microcentrifuge tube.

11. Following bead cleanup, quantify with a fluorometer or spectrophotometer. Validate enrichment by qPCR (Auxiliary protocol B). Enriched DNA can be sequentially enriched by repeating the enrichment protocol adding 30 µl of the enriched product to the FecalSeq enrichment protocol: step 17.

Auxiliary protocol B: qPCR estimation of enrichment

Materials and reagents

- Extracted fDNA of known quantity
- 2X SYBR Green master mix (e.g., Qiagen cat. #204143 or ThermoFisher Scientific cat. #A25780)
- Taxon-specific primers
- DNA standards
  For host quantification, standards can be created by performing a dilution series (i.e., 10 ng/µl, 1 ng/µl, 0.1 ng/µl, 0.01 ng/µl) of high-quality gDNA (such as blood or liver DNA) from a suitable taxon.
- qPCR instrument

Procedures

1. Run samples and standards at least in duplicate. We also recommend running a positive and negative control with each set of quantifications.

2. Use primers specific to the analysis

   a. The proportion of host DNA can be quantified by comparing qPCR results using host-specific primers to the absolute quantification estimated by some independent means (e.g., fluorometer or spectrophotometer). For our baboon DNA quantifications, we use universal mammal primers for the *MYCBP* (c-myc) gene (Morin et al. 2001):

   b. Enrichment of DNA captured with MBD beads can be quantified as above using host-specific primers with enriched methylated host DNA. Alternatively, enrichment can be estimated by observing the *n*-fold decrease in quantified levels from unenriched to enriched samples using the universal 16S rRNA primer (Corless et al. 2000). 1 μl of unenriched DNA can be diluted to the concentration of the enriched sample prior to qPCR to standardize concentrations. Because MBD enrichment can in principle be biased towards densely methylated areas of the host genome, we prefer the latter method for estimating enrichment success.

Primer ID	Type	Locus	Sequence	Reference
cmycF<br>cmycR	mammalian	*MYCBP*	GCCAGAGGAGGAACGAGCT<br>GGGCCTTTTCATTGTTTTCCA	Morin et al. 2001
16S_F<br>16S_R	bacterial	16S rRNA	CCATGAAGTCGGAATCGCTAG<br>GCTTGACGGGCGGTGT	Corless et al. 2000

3. Set up qPCR reactions in a 20 μl total volume containing 1X of SYBR Green master mix, 0.5 mM of each primer, and 1 μl of DNA.

4. Run samples in the qPCR instrument at 95 °C for 15 minutes, followed by 50 cycles of 94 °C for 15 seconds, 59 °C (for all primers specified above; adjust for other primers) for 25 seconds, and 72 °C for 20 seconds.

References

Corless CE, Guiver M, Borrow R, Edwards-Jones V, Kaczmarski EB, Fox AJ. 2000. Contamination and sensitivity issues with a real-time universal 16S rRNA PCR. *J Clin Microbiol* **38**: 1747–1752.

Gaillard C, Strauss F. 1998. Avoiding adsorption of DNA to polypropylene tubes and denaturation of short DNA fragments. *Tech Tips Online* **3**: 63–65.

Morin PA, Chambers KE, Boesch C, Vigilant L. 2001. Quantitative polymerase chain reaction analysis of DNA from noninvasive samples for accurate microsatellite genotyping of wild chimpanzees (*Pan troglodytes verus*). *Mol Ecol* **10**: 1835–1844.

Rohland N, Reich D. 2012. Cost-effective, high-throughput DNA sequencing libraries for multiplexed target capture. *Genome Res* **22**: 939–946.
